# 2D-NMR characterization of higher substituted oligosaccharides isolated from enzymatic wheat flour arabinoxylan hydrolysates

**DOI:** 10.3389/fpls.2026.1784230

**Published:** 2026-03-09

**Authors:** Lukas Sitter, Mirko Bunzel

**Affiliations:** Institute of Applied Biosciences, Department of Food Chemistry and Phytochemistry, Karlsruhe Institute of Technology (KIT), Karlsruhe, Germany

**Keywords:** arabinoxylooligosaccharides, cereal grain arabinoxylans, enzymatic hydrolysis, NMR, semi-preparative HILIC, standard compounds

## Abstract

Arabinoxylans (AXs) play a substantial role in the cell walls of cereals, contributing to their structural integrity and stability. The physicochemical and physiological properties of AX structures vary depending on the degree and pattern of substitution. AX structures are based on a linear β-(1→4)-linked d-xylopyranose backbone, being partially substituted with α-L-arabinofuranose in *O*2 and/or *O*3 positions of the xylopyranose units. Alkaline-extracted wheat flour AX were hydrolyzed with *endo*-β-1,4-xylanases of glycoside hydrolase (GH) families 10 or 11. The resulting arabinoxylooligosaccharides (AXOS) were isolated and purified using various chromatographic techniques, including gel permeation chromatography, semi-preparative hydrophilic interaction chromatography, and high performance anion exchange chromatography. The isolated, purified compounds were characterized by their monosaccharide composition, molecular weight, and monomer binding positions via one- and two-dimensional NMR experiments. In addition to smaller AXOS, higher-substituted oligosaccharides with consecutive mono- and disubstituted xylose residues were identified in the hydrolysates, proving that GH10 and GH11 *endo*-xylanases can cleave more densely substituted regions of wheat AX, in which disubstitution is preferred. These oligosaccharides were obtained in quantities and purities sufficient for their use as standard compounds. We report complete NMR data sets for the four most complex AXOS (A^2+3^XA^2+3^XX, A^2+3^A^2+3^XX, XA^3^A^2+3^XX, A^3^A^2+3^XX) for the first time and also provide a complete NMR library for 17 (A)XOS that were either isolated here or commercially available.

## 1 Introduction

Arabinoxylans (AXs) are among the dominant polysaccharides found in the cell walls of commelinid monocot plants (Vogel, 2008). This botanical group includes the family of Poaceae which contains cereals such as wheat, maize, barley, and rye. In the plant cell wall, AXs are closely associated with cellulose microfibrils and, in case of secondary cell wall formation, lignin, forming a variable and highly complex network. Through these interactions, AXs contribute to the plant’s structural integrity, increase its flexibility, and provide a protective barrier against insects, disease, and abiotic stressors (Simmons et al., 2016; Zhang et al., 2021). Additionally, AXs are considered dietary fiber and thus play an important role in the human diet. Their physiological effects, particularly pronounced prebiotic properties, are closely linked to the structure of AXs (Broekaert et al., 2011).

The structure of AXs is based on a linear (1→4)-linked β-d-xylopyranose (Xyl*p*) backbone. Various substituents can be attached to this backbone, with α-l-arabinofuranoses (Ara*f*) being the most abundant. Ara*f* units are commonly present as mono- and/or disubstitutions at the *O*-2 and/or *O*-3 positions of the Xyl*p* units. However, other backbone decorations such as glucuronic acid, its 4-*O*-methyl derivative, or acetyl groups are also observed, particularly in maize and sorghum arabinoxylans (Verbruggen et al., 1995, 1998; Huisman et al., 2000). Covalently attached hydroxycinnamic acids are additional key features of cereal AXs. In particular, *trans*-ferulic acid is bound via an ester-bond to the *O*-5 position of Ara*f*. Free-radical-induced oxidative cross coupling of ferulates stabilizes cereal grains’ cell walls by covalently cross-linking AXs to each other and to lignin. Cross coupling does not only have an effect on the cell wall’s stability but also on its microbial fermentation in the human large intestine during digestion (Hopkins et al., 2003; Bunzel, 2010).

Average AX contents in wheat grains vary among species, tissues, and milling fractions ranging from 1.7 - 2.0% in wheat flour and 8.9 - 18.0% in wheat bran (Gebruers et al., 2008). Wheat AX structures have been extensively researched in the past. Depolymerization of AXs into low molecular weight oligosaccharides by specific *endo*-β-(1→4)-xylanases, which cleave the internal β-(1→4)-linkages, is a common approach to analyze AX structures (Gruppen et al., 1992b, 1993; McCleary et al., 2015). AXs serve as substrate for enzymes from three different glycoside hydrolase (GH) families: GH5, GH10, and GH11, but most studies focus on the most prominent GH10 and GH11 xylanases (Biely et al., 2016; Mathew et al., 2017; Bhattacharya et al., 2020). GH10 xylanases are less hindered by backbone substitution than their GH11 counterparts and require only two consecutive unsubstituted xylose units for hydrolysis, compared to three for GH11 xylanases (Pollet et al., 2010). Both enzymes exhibit limited efficacy in degrading densely-substituted areas of AX. In GH10 hydrolysates, branched oligosaccharides usually retain their substituents at the non-reducing end, and the reducing end contains two unsubstituted units. However, this cleavage pattern is not exclusive, as arabinoxylooligosaccharides (AXOS) with only one unsubstituted Xyl*p* residue on the reducing end or with an additional Xyl*p* on the non-reducing end have also been detected after GH10 hydrolysis. AXOS, generated by GH11 xylanases, contain two unsubstituted Xyl*p* residues at the reducing end, as they do not tolerate substituents on the +1 subsite (Biely et al., 2016; Capetti et al., 2021).

Promising approaches to structurally characterize AXs in more detail are often based on enzymatic liberation of AXOS followed by their analysis using chromatographic techniques such as high performance anion-exchange chromatography with pulsed amperometric detection (HPAEC-PAD) (Ordaz-Ortiz et al., 2005; Joyce et al., 2023). However, these methods are often restricted since they are usually based on a limited number of commercially available standard compounds, representing only specific regions of the AX structure. Also, 2D-NMR of enzymatically or chemically liberated oligosaccharides can be used in polysaccharide profiling approaches, but also require standard compounds to define signals that are diagnostic for specific structural features and to perform calibration (Wefers and Bunzel, 2016; Schendel and Bunzel, 2022). Therefore, the aim of this work was to isolate non-commercial AXOS standard compounds from alkaline extracted wheat flour arabinoxylans after GH10 and GH11 hydrolysis and to obtain complete NMR data sets of these oligosaccharides.

## 2 Materials and methods

### 2.1 Chemicals and materials

Wheat flour AX (medium viscosity) and standard compounds of xylooligosaccharides (XOS) X_2_ - X_6_ and AXOS A^3^X, A^2^XX, A^2+3^XX, XA^2^XX/XA^3^XX (mixture in a ratio of 47: 53) were purchased from Megazyme (Wicklow, Ireland).

Ultra-pure water (Milli-Q, Millipore) was used for the preparation of aqueous solutions and chromatography eluents. Organic solvents of appropriate purity (e.g., ethanol, acetonitrile, or dichloromethane) and iodomethane for methylation analysis were from VWR (Darmstadt, Germany). Dimethyl sulfoxide, glacial acetic acid, sodium hydroxide (powder), and sodium thiosulfate were purchased from Carl Roth GmbH + Co. KG (Karlsruhe, Germany). Eluents for high performance ion exchange chromatography (HPAEC) were prepared with sodium acetate (Honeywell Fluka), 49 - 51% concentrated sodium hydroxide (NaOH) solution (ion chromatography grade, Sigma-Aldrich), and degassed water and were stored under helium pressure. Trifluoroacetic acid (TFA) (suitable for HPLC, purity ≥ 99.0%) and hydrochloric acid in 3 M methanol solution for monosaccharide analysis as well as 1-methylimdiazole were also obtained from Sigma-Aldrich Inc. (Darmstadt, Germany). Acetic anhydride was purchased from Honeywell Specialty Chemicals Seelze GmbH (Hannover, Germany). Deuterium oxide (D_2_O, 99.9% purity) for NMR measurements as well as sodium borodeuteride (NaBD_4_) were from Deutero GmbH (Kastellaun, Germany).

Recombinant GH10 *endo*-β-1,4-xylanase (EC 3.2.1.8, *Clostridium thermocellum*, Xylanase 10B) was purchased from Nzytech (Lisboa, Portugal). Recombinant GH11 *endo*-β-1,4-xylanase (EC 3.2.1.8, *Neocallimastix patriciarum*, E-XYLNP) was from Megazyme (Wicklow, Ireland).

### 2.2 Characterization of wheat flour arabinoxylan

#### 2.2.1 Monosaccharide composition by methanolysis

To analyze the monosaccharide composition of wheat flour AX, an aliquot was hydrolyzed in 500 µL of a hydrochloric acid in 3 M methanol solution for 16 h at 80°C. After evaporation, the residue was hydrolyzed in 500 µL of 2 M TFA for 1 h at 121°C (De Ruiter et al., 1992; Willför et al., 2009). Samples were evaporated again to dryness and co-evaporated twice with 200 µL of ethanol. The residues were redissolved in ultrapure water, and 2-deoxy-d-glucose was added to the solution as an internal standard compound (final concentration: 25 µM) for measurement. The samples were analyzed by analytical HPAEC-PAD on an ICS-5000 system (ThermoFisher Scientific Dionex, Sunnyvale, CA, USA) using a CarboPac PA-20 column (6 µm, 150 x 3 mm, ThermoFisher Scientific) at 25°C. A flow rate of 0.4 mL/min and the following gradient composed of ultrapure water (A), 0.1 M NaOH (B), and 0.1 M NaOH with 500 mM sodium acetate (C) as eluents were used: Before every run the column was rinsed with 100% B for 10 min and equilibrated with 90% A and 10% B for 10 min. After injection, the following linear gradient was applied: 0 - 1.5 min, from 90% A and 10% B to 97% A and 3% B; 1.5–22 min, isocratic at 97% A and 3% B; 22–27 min, from 97% A and 3% B to 100% B; 27.1 - 37.0 min, isocratic at 60% B and 40% C.

#### 2.2.2 Determination of binding positions by methylation analysis

Methylation analysis was carried out as described by Schendel et al. (2015) with minor modifications. Wheat flour AX (3 mg) was dissolved in 1 mL of dimethyl sulfoxide, to which ca. 50 mg of freshly ground sodium hydroxide was added. The mixture was incubated for 90 min in an ultrasonic bath and for additional 90 min at room temperature. Then, 0.5 mL of methyl iodide was added, followed by 30 min of sonication and 30 min of incubation at room temperature. The solution was extracted with dichloromethane, and the organic phase was washed with 2.5 mL of 0.1 M sodium thiosulfate, then twice with 1.5 mL of water. The solvent was evaporated, and the samples were dried overnight in a vacuum oven at 40°C. The methylated polysaccharides were hydrolyzed by adding 1 mL of 2 M TFA and incubating at 121°C for 90 min. After evaporation of the acid, 10 mg of NaBD_4_ in 150 µL of a 2 M aqueous ammonia solution was added. Reduction was carried out at room temperature for 60 min and terminated by the addition of glacial acetic acid. While cooling on ice, 225 μL of 1-methylimidazole and 1.5 mL of acetic anhydride were added. The solution was then incubated for 30 min at room temperature. After adding 1.5 mL of water, the solution was extracted with 2.5 mL of dichloromethane. The organic layer was washed three times with water, and the residual water was removed by freezing overnight at −18°C. Gas chromatography-mass spectrometry (GC-MS) analysis of the partially methylated alditol acetates (PMAAs) was performed using GC-2010 Plus and GCMS-QP2010 SE instruments (Shimadzu, Kyoto, Japan), which were equipped with a DB-5 MS column (30 m × 0.25 mm inner diameter [i.d.], 0.25 μm film thickness) (Agilent Technologies, Santa Clara, CA). The following conditions were used: The initial column temperature was 140°C, held for two minutes, then increased to 180°C at a rate of 1°C/min, and held for five minutes. This was followed by an increase to 300°C at a rate of 10°C/min. Helium was used as the carrier gas at 40 cm/s. Split injection with a split ratio of 30:1 was used, and the injection temperature was 220°C. The transfer line temperature was 220°C, and electron impact mass spectra were recorded at 70 eV. PMAAs were quantified by analyzing the samples by GC-FID (GC-2010 Plus) (Shimadzu) using the same conditions as described above but applying a 10:1 split ratio. Nitrogen was used as makeup gas, and the FID temperature was 240°C. Molar response factors according to Sweet et al. (1975) were used for a semiquantitative determination. The analysis was performed in duplicate.

### 2.3 Enzymatic degradation of wheat flour arabinoxylan by *endo*-β-1,4-xylanases

Two incubation batches were carried out for each enzyme. Wheat flour AX (1 g) was weighed into an Erlenmeyer flask and dissolved in ultrapure water (80 mL) while stirring on a hotplate. Recombinant GH10 and GH11 *endo*-β-1,4-xylanases were added to the respective batches at a concentration of 7 U/mL (GH10) and 500 U/mL (GH11). The GH10 mixture was incubated for 4 h at 75°C and the GH11 mixture for 4 h at 50°C. Incubations were stopped by adding ethanol to a final concentration of 60% (v/v), and undigested, precipitated polysaccharides were removed by centrifugation. The supernatants were concentrated on a rotary evaporator (water bath temperature: 50°C) and finally freeze-dried.

### 2.4 Isolation and clean-up of arabinoxylan oligosaccharides

#### 2.4.1 Gel permeation chromatography

Freeze-dried hydrolysates were redissolved in ultrapure water (20 mL) and fractionated by using Bio-Gel P2 chromatography (Bio-Rad Laboratories GmbH, Feldkirchen, Germany) (sample loop size: 10 mL, bed volume: 85 x 2.6 cm). Elution was carried out with ultrapure water (0.5 mL/min) at 45°C, and the effluent was monitored by refractive index detection (Smartline RI detector 2300, Knauer, Berlin, Germany). AXOS-containing fractions were collected in intervals of 6 min and combined according to the chromatogram.

#### 2.4.2 Semi-preparative HILIC chromatography

Gel permeation chromatography fractions of the GH10 hydrolysate and the GH11 hydrolysate were further purified on either a porous graphitized carbon (PGC) column (Hypercarb, 100 × 4.6 mm, 5 μm particle size, Thermo Fisher Scientific) or a hydrophilic interaction liquid chromatography (HILIC) column (XBridge BEH Amide, 250 mm x 10 mm, 5 µm particle size, Waters GmbH, Eschborn, Germany) using semipreparative high-performance liquid chromatography (HPLC) equipment (Azura P2.1L, Knauer Wissenschaftliche Geräte GmbH, Berlin, Germany). The eluent was split, and 1/30 was directed to an evaporative light scattering detector (ELSD, Sedex 85, ERC GmbH, Riemerling, Germany). The larger portion was collected according to the respective peaks in the chromatogram by using a programmable multiposition valve (Azura V2.1S, KNAUER). Individually adjusted gradient programs were applied to purify each AXOS using water and acetonitrile (PGC and HILIC) as eluents at 70°C (PGC) or 50°C (HILIC) and flow rates of 1–4 mL/min.

Additional purification was achieved by using semipreparative HPAEC-PAD on an ICS-5000 system. Aliquots of the fractions (injection volume: 100 µL) were separated on a CarboPac PA-100 column (8.5 µm, 250 x 9 mm, ThermoFisher Scientific) at 25°C using ultrapure water (A), 0.1 M NaOH (B), and 0.1 M NaOH with 500 mM sodium acetate (C) as eluents; the flow rate was 2 mL/min. Gradient programs had to be individually adapted to the specific AXOS. The eluent was split, and 1/20 was directed to the PAD. The larger portion was collected by using an UltiMate AFC-3000 multiposition fraction collector (Thermo Scientific). The fractions were neutralized with 0.1 M hydrochloric acid and desalted using non-PGC-stationary phase extraction (250 mg, 6 mL, Supelclean ENVI-Carb, Sigma Aldrich), which contained the following steps: conditioning with acetonitrile (3 x 1 mL) and ultrapure water (3 x 1 mL), stepwise loading of the sample, washing with ultrapure water (4 x 1 mL), elution of the oligosaccharides with acetonitrile/ultrapure water (50/50, 3 x 1 mL), reconditioning with acetonitrile (4 x 1.5 mL), ultrapure water (4 x 1.5 mL), and acetonitrile (2 x 1.5 mL), repeating the cycle. The eluates were finally evaporated and freeze-dried.

### 2.5 Characterization of isolated arabinoxylooligosaccharides

#### 2.5.1 HPAEC-PAD analysis of isolated arabinoxylooligosaccharides

The purity of the isolated standard compounds was assessed through analytical HPAEC-PAD on an ICS-5000 system. Solutions of the standard compounds were diluted and aliquots (injection volume: 25 µL) were separated on a CarboPac PA-200 column (6 µm, 250 x 3 mm, ThermoFisher Scientific) at 25°C using the same eluents as described in section 2.2.1; the flow rate was 0.4 mL/min. Before every run the column was rinsed with 100% C for 10 min and equilibrated with 90% A and 10% B for 20 min. After injection, the following gradient was applied: 0–10 min, from 90% A and 10% B to 35% A and 65% B; 10–18 min, from 35% A and 65% B to 35% A, 63.5% B, and 1.5% C; 18–22 min, isocratic; 22–60 min, from 35% A, 63.5% B, and 1.5% C to 31% A, 53% B, and 16% C; 60–80 min, from 31% A, 53% B, and 16% C to 27% A, 49% B, and 24% C; 80.1–90 min, isocratic with 100% C. Purity was calculated by integrating all peaks in the chromatogram including the standard compound. By using this analysis, nine AXOS were deemed sufficiently pure (> 90%) following the separation procedure described above.

#### 2.5.2 Monosaccharide composition

To obtain information on the arabinose-to-xylose-ratio of the individual AXOS the monosaccharide composition was analyzed. Aliquots were evaporated, and AXOS were hydrolyzed using 500 µL of 2 M TFA for 1 h at 121°C. Samples were evaporated to dryness and co-evaporated twice with 200 µL of ethanol. The residues were redissolved in ultrapure water and 2-deoxy-d-glucose was added to the solution as an internal standard compound (final concentration: 25 µM) for measurement. The samples were analyzed by analytical HPAEC-PAD on an ICS-5000 system using a CarboPac PA-20 column (6 µm, 150 x 3 mm) at 25°C. A flow rate of 0.4 mL/min and the gradient already described in section 2.2.1 was used.

#### 2.5.3 Molecular weight determination by HPAEC-PAD/MS

HPAEC-PAD coupled to a mass spectrometer (MS) based analysis was performed on an ICS-6000 system (ThermoFisher Scientific Dionex) equipped with an analytical CarboPac 200 column (see section 3.5.1). To enable simultaneous PAD and MS analysis, the eluent was split into two parts (ratio 1 to 2), and the smaller part was analyzed using PAD. Prior to MS analysis, the eluent was desalted using an electrolytically regenerated suppressor (AERS 500, ThermoFisher Scientific), and 50 µM lithium chloride solution was added (flow rate: 0.05 mL/min, AXP-MS pump, ThermoFisher Scientific) for the formation of characteristic lithium adducts during electrospray ionization in order to obtain the molecular weight of the individual standard compounds. Chromatographic conditions were described in section 2.5.1.

#### 2.5.4 One- and two-dimensional NMR characterization

Purified and freeze-dried AXOS (1–3 mg) were dissolved in 550 µL of D_2_O and 0.5 µL of acetone was added to the solution as an internal reference (δ_H/C_ = 2.22/30.89 ppm) for nuclear magnetic resonance (NMR) spectroscopy (Gottlieb et al., 1997). However, this was also dependent on the available quantities of the respective AXOS, meaning that the entire quantity obtained had to be used for some standard compounds. Complete structural elucidation was accomplished by NMR spectroscopy using a Bruker Ascend 500 MHz spectrometer (Bruker corporation, Rheinstetten, Germany) equipped with a Prodigy cryoprobe operating at 298 K. Standard Bruker pulse sequences of ^1^H (zg30), phase-sensitive HSQC (hsqcedetgp), HMBC (hmbcgplpndqf), HSQC-TOCSY (hsqcetgpml), and H2BC (h2bcetgpl3) experiments were applied to each standard compound. In order to reduce experiment time, nonuniform sampling (50% sampling density) was used in all experiments. After NMR-analysis, the standard compounds could easily be recovered by freeze-drying.

## 3 Results

### 3.1 General characterization of wheat flour arabinoxylans

The monosaccharide composition of the alkaline extracted wheat flour AX was 32.07 ± 0.20 mol% arabinose and 67.93 ± 0.20 mol% xylose. The arabinose/xylose ratio of 0.47 demonstrated a low degree of substitution along the AX backbone. This is consistent with data published by Gruppen et al. (1992a), who reported an arabinose/xylose ratio of 0.52 in alkaline extracted wheat flour AXs. Methylation analysis provided further structural insights into wheat flour AX (see **Table 1**). The arabinose/xylose ratio (0.41) calculated across all linkage types, that is, the sum of Ara*f* (28.83 ± 0.46 mol%) and Xyl*p* (70.80 ± 1.45 mol%) units, was comparable to the value obtained from the monosaccharide analysis. Nearly 60% of the xylose units are unsubstituted. Among the remaining xylose residues from wheat flour AX, roughly one half was monosubstituted, while the other half was disubstituted. Substitution patterns of wheat AXs vary depending not only on the wheat species but also on the tissue type and the extraction method used. Barium hydroxide extracted insoluble wheat bran AX shows a comparable substitution pattern with 53% of the Xyl*p* units being unsubstituted, 20% monosubstituted and 18% disubstituted. Also the *O*3/*O*2 ratio (11.4) was in the range of the wheat flour AX (9.5) (Schooneveld-Bergmans et al., 1999). Most of the arabinose units (92%) were terminal, but some were substituted, suggesting the presence of oligosaccharide side chains attached to the arabinoxylan backbone. Oligosaccharides containing (1→2)-linked arabinobiose side chains have been isolated from sorghum and switchgrass biomass (Verbruggen et al., 1998; Mazumder and York, 2010). However, it is more likely that the *O*-2 substituted arabinose originates from feruloylated side chains containing an additional xylose substituent, which has been detected in various cereal grains (Schendel et al., 2016). 3-Ara*f* likely originates from non-feruloylated α-d-Xyl*p*-(1→3)-L-arabinose disaccharide side-chains (Schendel and Bunzel, 2025). Structures containing (1→5)-glycosidically bound Ara*f* potentially stem from oligomeric side-chains (Izydorczyk and Biliaderis, 1993), but these side-chains have not been unambiguously proven to date. The presence of small amounts of 1,3- and 1,4-linked glucose suggests that alkaline extractable mixed-linked β-glucans are also present. Like arabinoxylans, mixed-linked β-glucans are widespread in grasses, but usually minor in wheat (Lazaridou and Biliaderis, 2007).

**Table 1:** Methylation analysis data of wheat flour arabinoxylan.

Linkage type	Mean^a^	Range/2
t-Ara*f*	26.48	0.34
1,2-Ara*f*	1.29	0.03
1,3-Ara*f*	0.18	0.04
1,5-Ara*f*/1,4-Ara*p*	0.66	0.03
1,2,3,5-Ara*f*	0.22	0.02
**Σ Ara*f***	**28.83**	**0.46**
t-Xyl*p*	2.13	0.27
1,4-Xyl*p*	39.93	0.88
1,2,4-Xyl*p*	1.37	0.19
1,3,4-Xyl*p*	13.05	0.06
1,2,3,4-Xyl*p*	14.31	0.05
**Σ Xyl*p***	**70.80**	**1.45**
t-Glc*p*	0.08	0.01
1,3-Glc*p*	0.12	0.01
1,4-Glc*p*	0.12	0.01
**Σ Glc*p***	**0.32**	**0.03**
t-Gal*p*	0.05	0.01

a n = 2; values are presented as relative molar percentages.

Ar*af*, arabinofuranose; Gal*p*, galactopyranose; Xyl*p*, xylopyranose.

Bold values represent the sum of all linkage types for a specific monosaccharide.

### 3.2 Isolation of arabinoxylooligosaccharides from wheat flour arabinoxylan

**Figure 1:**
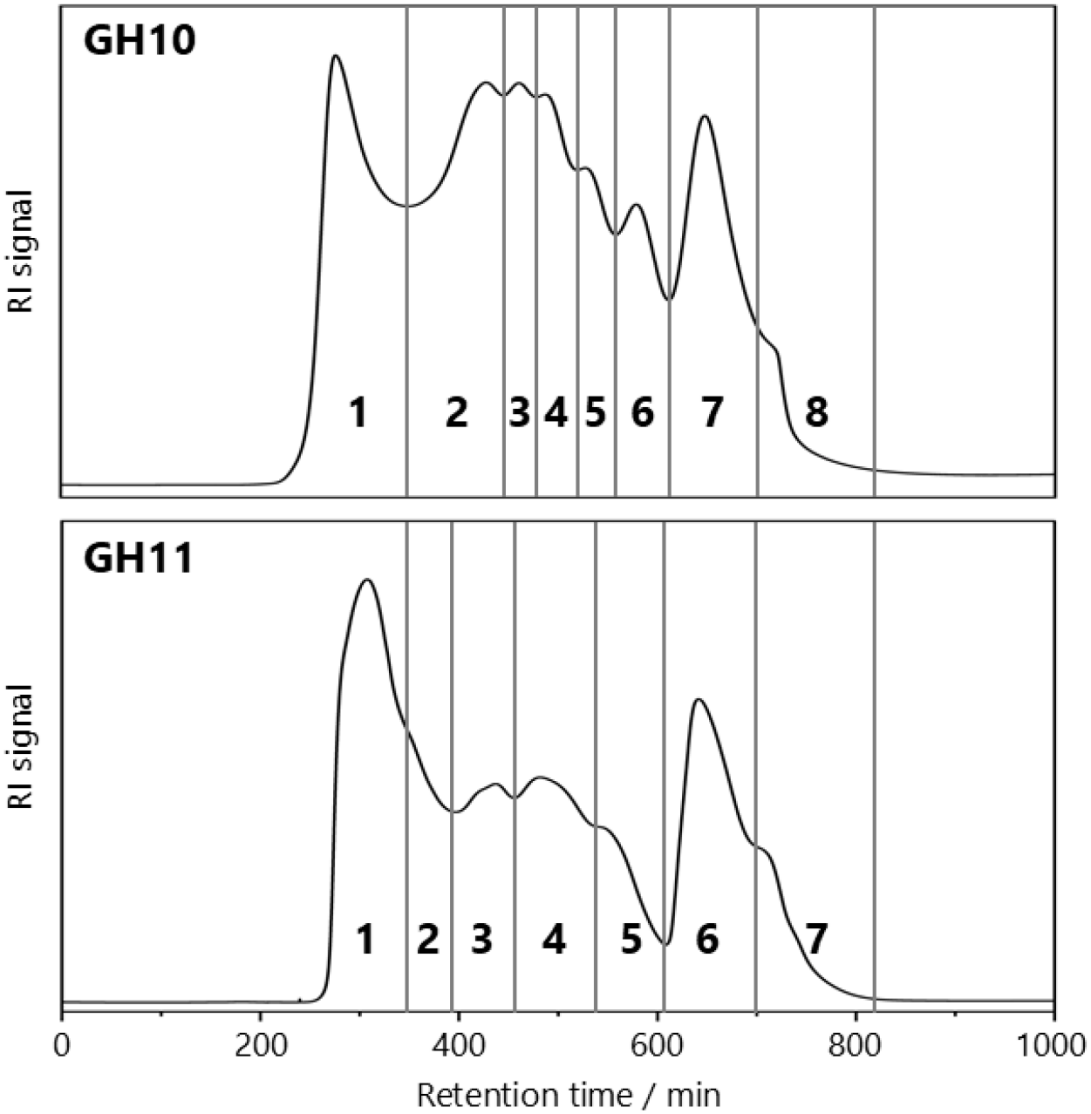
BioGel P-2 separation of GH10 (top) and GH11 hydrolysates (bottom); RI, refractive index detection.

**Figure 2:**
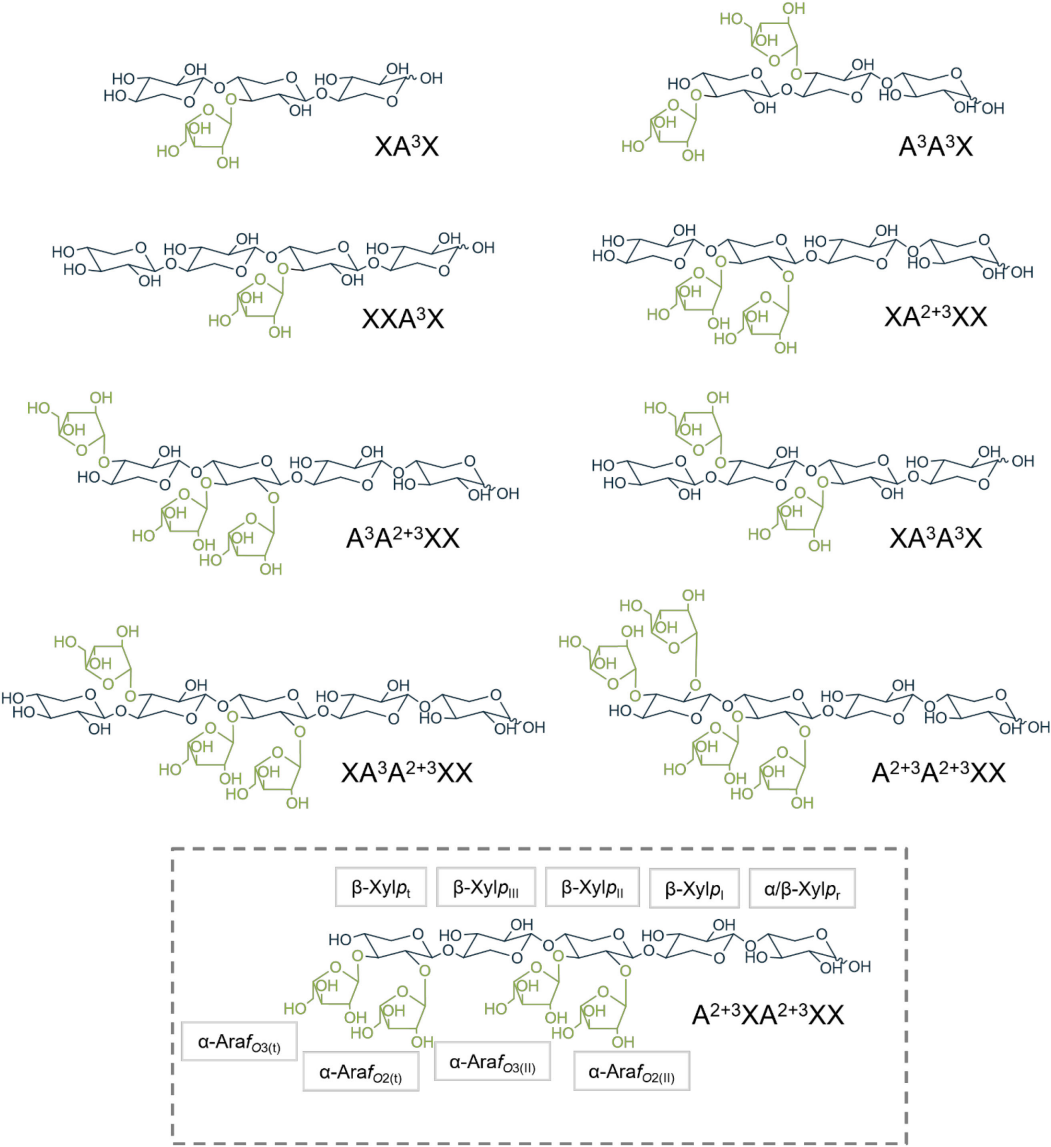
Arabinoxylooligosaccharide structures isolated from enzymatic hydrolysates of wheat flour arabinoxylan. The abbreviated structural names are taken from the naming system suggested by Fauré et al. (2009). The positions of the xylopyranose (Xyl*p*) units (marked in gray) in the molecule are labeled in subscript from right to left, starting from the reducing end (r) to the terminal end (t) with Roman numbers (I-III). For arabinofuranoses (Ara*f*, marked in green), the subscript indicates the binding position to the corresponding Xyl*p* unit (in parentheses).

Two complementary incubation batches were performed with *endo*-xylanases of either the GH10 or the GH11 family, which both cleave β-(1→4)-glycosidic linkages exclusively within the xylose backbone chain of the arabinoxylan but in different positions (Pollet et al., 2010). While GH11 xylanases require three consecutive unsubstituted xylose units, GH10 xylanases only require two (Biely et al., 2016). The wheat flour arabinoxylan was completely degraded to low-molecular-weight oligosaccharides as no residue remained after ethanol precipitation. **Figure 1** shows gel permeation (BioGel P-2) chromatograms of the enzymatic hydrolysates. Both chromatograms are comparable but differ in detail. GH11 hydrolysis resulted in a reduced number of fractions (GH10: 8 fractions, GH11: 7 fractions), and the relative proportion of higher molecular weight AXOS that could not be further cleaved is greater than with GH10. This is likely due to the fact that there are fewer cleavage sites for GH11 xylanases than for GH10 (Pollet et al., 2010). Semi-preparative PGC, HILIC, and HPAEC were used to further purify the BioGel P-2 fractions, yielding nine AXOS standard compounds in addition to those that were already commercially available. Whether PGC separation was sufficient for isolation of an AXOS standard compound or whether HILIC and/or HPAEC was necessary in addition to the prior PGC separation was dependent on the BioGel fraction. All three chromatographic techniques are suitable for oligosaccharide separation, whether or not it works is not predictable. Thus, no clear preference for either separation mechanism was determined. The majority of AXOS standard compounds could only be purified to sufficient purity by applying two separation mechanisms. For a small number of individual fractions, HPAEC was additionally required. HPAEC usually provides substantially better resolution but requires subsequent desalting. The applied chromatographic purification steps for the different AXOS are provided in the supplementary data (**Supplementary Table 1**). The purity of the AXOS was greater than 90%, as determined by HPAEC-PAD, and gravimetrically determined amounts were in the range of one to nine mg. Their structures are shown in **Figure 2** using the naming system of Fauré et al. (2009), in which “X” stands for an unsubstituted Xyl*p* unit and “A” stands for a Xyl*p* unit substituted with Ara*f*. The superscript numbers indicate the respective binding position of the arabinose units. Accordingly, XA^3^X was obtained from BioGel fraction 5 of the GH10 hydrolysate. BioGel fraction 4 yielded XXA^3^X, XA^2+3^XX, and A^3^A^3^X, whereas fraction 3 contained XA^3^A^3^X, XA^2+3^XX, and A^3^A^2+3^XX; the latter was also detected in fraction 2. In addition, fraction 2 gave rise to more highly substituted AXOS, including A^2+3^A^2+3^XX and A^2+3^XA^2+3^XX. From the GH11 hydrolysate, only fraction 3 of the GH11 hydrolysate was subjected to further purification, yielding XA^3^A^2+3^XX.

### 3.3 Structural elucidation of the isolated standard compounds

The isolated and purified AXOS compounds were structurally elucidated taking into account their monomer compositions, mass-to-charge ratios (*m*/*z*), and NMR spectroscopic data, the latter being presented in **Tables 2**–**5**. Assignments made were in accordance with previously published data, if available (Gruppen et al., 1992b; McCleary et al., 2015). The HSQC spectra of the individual, isolated standard compounds can be found in the supplementary data (**Supplementary Figures 1-9**). Additionally, the supplementary material also includes NMR data (**Supplementary Table 2**) and HSQC spectra (**Supplementary Figures 10-18**) of commercially available XOS and AXOS standard compounds.

**Table 2:** ^1^H and ^13^C chemical shifts of the isolated arabinoxylooligosaccharides (AXOS) with four to five pentose units.

Unit	H1	H2	H3	H4	H5
	C1	C2	C3	C4	C5
XA^3^X					
α-Xyl*p*_r_	5.18 (3.72)	3.75	3.54	3.75	3.81/3.75
	92.67	71.57	72.01	77.25	59.44
β-Xyl*p*_r_	4.58 (7.85)	3.25	3.54	3.78	4.05/3.36
	97.11	74.63	74.55	77.08	63.60
β-Xyl*p*_I_	4.51 (7.72)	3.44	3.74	3.83	4.12/3.41
	102.26	73.91	77.77	74.23	63.43
β-Xyl*p*_t_	4.44 (7.82)	3.24	3.41	3.59	3.91/3.27
	102.10	73.58	76.24	69.80	65.75
α-Ara*f*_O3(I)_	5.39	4.16	3.90	4.27	3.79/3.72
	108.22	81.32	77.85	85.43	61.94
XXA^3^X					
α-Xyl*p*_r_	5.18 (3.69)	3.75	3.55	3.75	3.82/3.75
	92.68	71.57	72.05	77.29	59.46
β-Xyl*p*_r_	4.59 (7.84)	3.25	3.54	3.78	4.05/3.37
	97.11	74.63	74.55	77.08	63.59
β-Xyl*p*_I_	4.52 (7.77)	3.44	3.74	3.83	4.11/3.41
	102.26	73.91	77.93	74.31	63.43
β-Xyl*p*_II_	4.47 (7.52)	3.27	3.54	3.75	4.04/3.35
	101.94	73.58	74.31	76.97	63.52
β-Xyl*p*_t_	4.45 (7.65)	3.25	3.42	3.62	3.97/3.29
	102.42	73.42	76.24	69.80	65.85
α-Ara*f*_O3(I)_	5.39	4.16	3.91	4.27	3.81/3.78
	108.30	81.40	77.85	85.34	61.90
A^3^A^3^X					
α-Xyl*p*_r_	5.18 (3.67)	3.54	3.75	3.75	3.81/3.74
	92.66	72.01	71.57	77.27	59.45
β-Xyl*p*_r_	4.59 (7.83)	3.25	3.54	3.78	4.05/3.37
	97.14	74.63	74.55	77.09	63.62
β-Xyl*p*_I_	4.51 (7.76)	3.44	3.74	3.83	4.12/3.83
	102.26	73.95	77.85	74.27	62.43
β-Xyl*p*_t_	4.48 (7.91)	3.40	3.57	3.66	3.94/3.31
	101.98	73.70	82.12	68.43	65.57
α-Ara*f*_O3(I)_	5.39	4.15	3.89	4.27	3.80/3.72
	108.26	81.36	77.85	85.41	61.94
α-Ara*f*_O3(t)_	5.32	4.17	3.95	4.18	3.81/3.71
	108.78	81.84	77.13	84.62	61.86

The vicinal couplings constants (^3^*J*_H,H_) to the neighboring proton is given in Hz in parentheses. The nomenclature of the AXOS is taken from the naming system according to Fauré et al. (2009). Abbreviations correspond to those shown in **Figure 2**.

**Table 3:** ^1^H and ^13^C chemical shifts of the isolated arabinoxylooligosaccharides (AXOS) with six to seven pentose units.

Unit	H1	H2	H3	H4	H5
	C1	C2	C3	C4	C5
XA^2+3^XX					
α-Xyl*p*_r_	5.18 (3.67)	3.54	3.74	3.74	3.81/3.74
	92.63	72.01	71.57	77.21	59.45
β-Xyl*p*_r_	4.58 (7.81)	3.25	3.54	3.77	4.04/3.37
	97.14	74.63	74.55	77.01	63.59
β-Xyl*p*_I_	4.46 (7.99)	3.29	3.56	3.79	4.14/3.41
	102.36	73.34	74.35	76.36	63.59
β-Xyl*p*_II_	4.63 (7.12)	3.57	3.82	3.86	4.14/3.43
	100.49	79.22	78.18	74.39	63.17
β-Xyl*p*_t_	4.43 (7.76)	3.25	3.41	3.60	3.92/3.27
	102.02	73.66	76.24	69.88	65.73
α-Ara*f*_O2(II)_	5.22	4.14	3.95	4.12	3.80/3.72
	109.35	81.84	77.31	85.06	61.86
α-Ara*f*_O3(II)_	5.27	4.16	3.93	4.30	3.78/3.73
	108.72	81.60	77.81	84.94	61.72
XA^3^A^3^X					
α-Xyl*p*_r_	5.18 (3.59)	3.54	3.75	3.74	3.82/3.74
	92.67	72.05	71.57	77.29	59.44
β-Xyl*p*_r_	4.58 (7.89)	3.24	3.54	3.78	4.05/3.37
	97.11	74.63	74.55	77.13	63.61
β-Xyl*p*_I_	4.51 (7.80)	3.44	3.73	3.83	4.12/3.39
	102.26	73.91	77.85	74.31	63.44
β-Xyl*p*_II_	4.49 (7.97)	3.43	3.73	3.79	4.06/3.39
	101.94	74.22	77.85	74.15	63.41
β-Xyl*p*_t_	4.43 (7.91)	3.23	3.40	3.59	3.90/3.28
	102.06	73.58	76.24	69.80	65.74
α-Ara*f*_O3(I)_	5.39	4.15	3.90	4.27	3.79/3.72
	108.22	81.35	77.86	85.43	61.94
α-Ara*f*_O3(II)_	5.38	4.15	3.90	4.27	3.79/3.72
	108.30	81.35	77.86	85.43	61.94
A^3^A^2+3^XX					
α-Xyl*p*_r_	5.18 (3.69)	3.54	3.75	3.74	3.81/3.74
	92.67	71.97	71.57	77.21	59.44
β-Xyl*p*_r_	4.58 (7.97)	3.24	3.54	3.77	4.04/3.37
	97.15	74.63	74.46	77.04	63.59
β-Xyl*p*_I_	4.46	3.29	3.56	3.79	4.14/3.41
	102.34	73.34	74.39	76.40	63.59
β-Xyl*p*_II_	4.64 (7.16)	3.57	3.82	3.87	4.14/3.43
	100.49	79.22	78.21	74.47	63.19
β-Xyl*p*_t_	4.46 (7.92)	3.40	3.58	3.67	3.95/3.30
	101.94	73.74	82.20	68.50	65.53
α-Ara*f*_O2(II)_	5.22	4.14	3.95	4.12	3.81/3.72
	109.35	81.84	77.29	85.06	61.82
α-Ara*f*_O3(II)_	5.26	4.16	3.93	4.30	3.79/3.71
	108.78	81.64	77.77	84.94	61.74
α-Ara*f*_O3(t)_	5.32	4.17	3.95	4.18	3.81/3.72
	108.78	81.88	77.13	84.62	61.82

The vicinal couplings constants (^3^*J*_H,H_) to the neighboring proton is given in Hz in parentheses. The nomenclature of the AXOS is taken from the naming system according to Fauré et al. (2009). Abbreviations correspond to those shown in **Figure 2**.

**Table 4:** ^1^H and ^13^C chemical shifts of the isolated arabinoxylooligosaccharides (AXOS) with eight pentose units.

Unit	H1	H2	H3	H4	H5
	C1	C2	C3	C4	C5
XA^3^A^2+3^XX					
α-Xyl*p*_r_	5.18 (3.66)	3.55	3.75	3.74	3.82/3.74
	92.68	72.05	71.57	77.21	59.44
β-Xyl*p*_r_	4.58 (7.83)	3.25	3.55	3.77	4.05/3.37
	97.11	74.63	74.47	77.05	63.62
β-Xyl*p*_I_	4.46 (7.75)	3.29	3.55	3.79	4.14/3.41
	102.34	73.34	74.39	76.40	63.59
β-Xyl*p*_II_	4.64 (7.22)	3.56	3.84	3.87	4.14/3.43
	100.49	79.23	78.20	74.55	63.19
β-Xyl*p*_III_	4.47 (7.64)	3.44	3.73	3.83	4.12/3.83
	101.86	74.31	77.85	74.27	62.43
β-Xyl*p*_t_	4.43 (7.91)	3.23	3.40	3.59	3.26/3.89
	102.02	73.59	76.24	69.80	65.73
α-Ara*f*_O2(II)_	5.22	4.14	3.95	4.12	3.81/3.72
	109.35	81.80	77.29	85.10	61.82
α-Ara*f*_O3(II)_	5.26	4.16	3.93	4.30	3.79/3.71
	108.77	81.64	77.85	84.94	61.74
α-Ara*f*_O3(III)_	5.40	4.15	3.90	4.27	3.80/3.72
	108.22	81.32	77.93	85.43	61.94
A^2+3^A^2+3^XX					
α-Xyl*p*_r_	5.17 (3.68)	3.74	3.54	3.74	3.81/3.73
	92.63	71.57	72.01	77.17	59.45
β-Xyl*p*_r_	4.58 (7.84)	3.24	3.54	3.76	4.04/3.37
	97.14	74.63	74.55	77.01	63.59
β-Xyl*p*_I_	4.46 (7.75)	3.28	3.55	3.78	4.13/3.41
	102.38	73.34	74.39	76.32	63.59
β-Xyl*p*_II_	4.62 (7.19)	3.56	3.85	3.85	4.13/3.48
	100.61	79.46	78.05	74.50	63.35
β-Xyl*p*_t_	4.54 (7.72)	3.54	3.66	3.68	3.95/3.29
	100.61	78.77	83.13	68.71	65.41
α-Ara*f*_O2(II)_	5.22	4.14	3.95	4.12	3.82/3.72
	109.39	81.84	77.29	85.02	61.82
α-Ara*f*_O3(II)_	5.29	4.16	3.93	4.33	3.82/3.72
	108.62	81.56	77.89	85.14	61.82
α-Ara*f*_O2(t)_	5.25	4.14	3.97	4.16	3.82/3.72
	109.23	81.91	77.36	84.94	61.82
α-Ara*f*_O3(t)_	5.24	4.17	3.97	4.19	3.82/3.72
	109.11	81.80	77.13	84.58	61.82

The vicinal couplings constants (^3^*J*_H,H_) to the neighboring proton is given in Hz in parentheses. The nomenclature of the AXOS is taken from the naming system according to Fauré et al. (2009). Abbreviations correspond to those shown in **Figure 2**.

**Table 5:** ^1^H and ^13^C chemical shifts of the isolated arabinoxylooligosaccharide (AXOS) with nine pentose units.

Unit	H1	H2	H3	H4	H5
	C1	C2	C3	C4	C5
A^2+3^XA^2+3^XX					
α-Xyl*p*_r_	5.18 (3.69)	3.74	3.54	3.74	3.81/3.74
	92.59	71.57	72.01	77.13	59.41
β-Xyl*p*_r_	4.58 (7.85)	3.24	3.54	3.76	4.11/3.38
	97.10	74.63	74.47	77.04	63.59
β-Xyl*p*_I_	4.46 (7.82)	3.29	3.55	3.78	4.11/3.38
	102.34	73.34	74.39	76.40	63.59
β-Xyl*p*_II_	4.63 (7.05)	3.56	3.82	3.86	4.13/3.42
	100.49	79.22	78.25	74.39	63.19
β-Xyl*p*_III_	4.43 (7.81)	3.28	3.55	3.75	4.11/3.38
	101.94	73.58	74.39	76.56	63.59
β-Xyl*p*_t_	4.58 (7.85)	3.53	3.68	3.71	4.01/3.32
	100.65	78.73	82.85	68.67	65.45
α-Ara*f*_O2(II)_	5.23	4.14	3.95	4.12	3.81/3.72
	109.35	81.88	77.29	85.02	61.82
α-Ara*f*_O3(II)_	5.27	4.16	3.93	4.30	3.81/3.72
	108.78	81.64	77.86	84.94	61.82
α-Ara*f*_O2(t)_	5.23	4.14	3.95	4.12	3.81/3.72
	109.35	81.88	77.29	85.02	61.82
α-Ara*f*_O3(t)_	5.24	4.17	3.97	4.19	3.81/3.72
	109.11	81.77	77.16	84.62	61.82

The vicinal couplings constants (^3^*J*_H,H_) to the neighboring proton is given in Hz in parentheses. The nomenclature of the AXOS is taken from the naming system according to Fauré et al. (2009). Abbreviations correspond to those shown in **Figure 2**.

**Figure 3:**
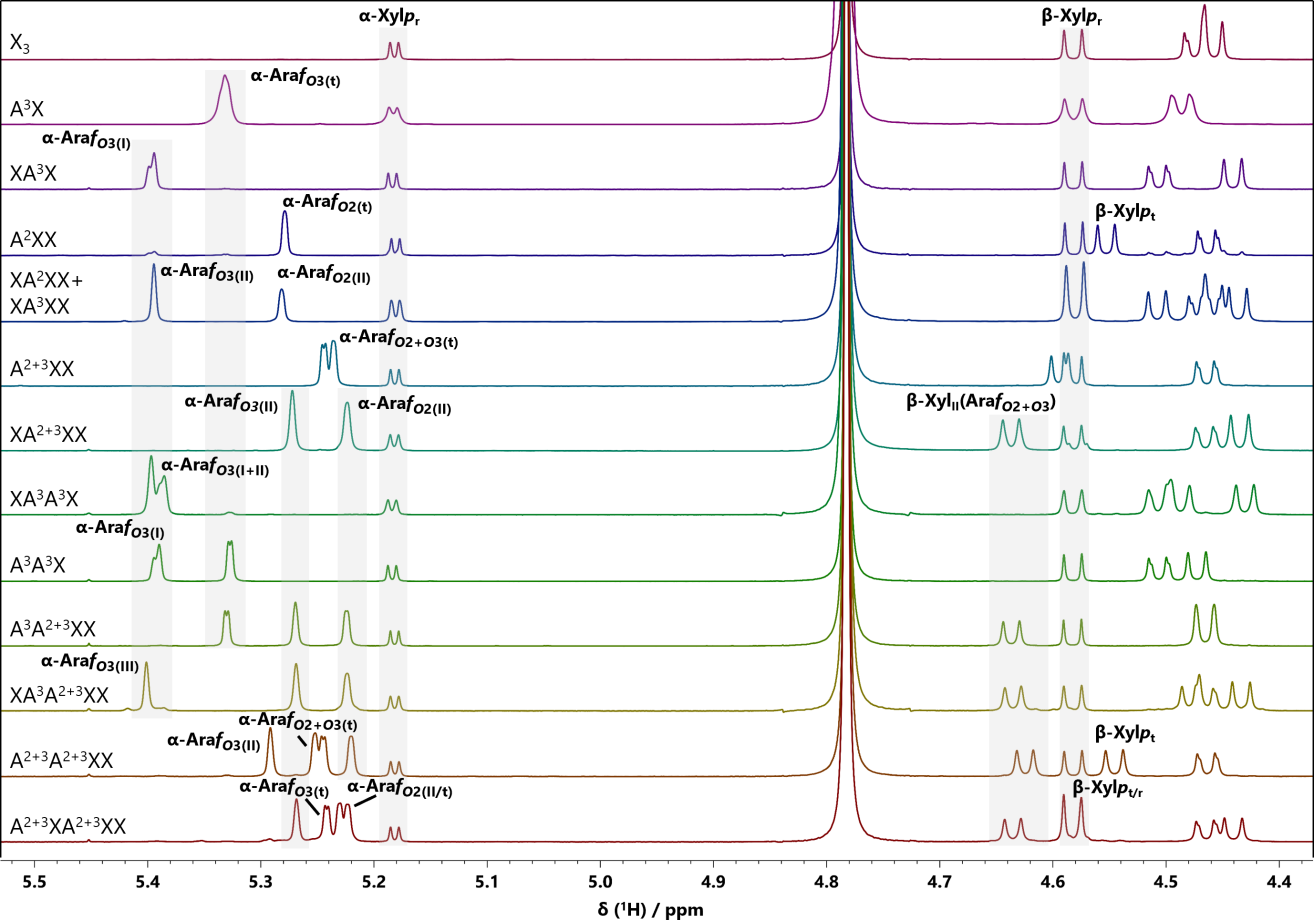
Superimposed one-dimensional ^1^H NMR spectra of selected xylooligosaccharides (XOS) and arabinoxylooligosaccharides (AXOS) in the range of the anomeric proton signals. The spectra were recorded in D_2_O on a 500 MHz spectrometer and calibrated against the chemical shift of acetone (δ_H_= 2.22 ppm). Complete spectra of these standard compounds are shown in the supplemental data (**Supplementary Figure 19**). The nomenclature of the standard compounds is taken from the naming system according to Fauré et al. (2009). Abbreviations correspond to those shown in **Figure 2**. Assigned chemical shifts are listed in **Tables 2**–**5** and **Supplementary Table 2**.

XA^3^X is the primary AXOS found in the GH10 hydrolysates of the wheat flour AX with gravimetrically determined amounts up to 9 mg after the isolation process. TFA hydrolysis of XA^3^X revealed a monosaccharide composition of xylose and arabinose in a molar ratio of 73.1 to 26.9. Additionally, the HPAEC-PAD/MS analysis clearly showed the presence of a lithium adduct with *m*/*z* 553, suggesting an X_3_ oligosaccharide with one arabinose. Several NMR experiments were applied in D_2_O to clearly elucidate the structure of the compound, including the identification of binding positions. The monomeric components of XA^3^X were confirmed by examining the ^1^H NMR spectrum (see **Figure 3**). Signals of anomeric protons are generally shifted to higher frequencies (4.40 - 5.50 ppm). Their vicinal coupling constants ^3^*J*_HH_ depend on the dihedral angle between H1 and H2, indicating their α- or β-configuration (Karplus equation) (Karplus, 1963). The reducing end of AXOS always consists of an unsubstituted xylose unit, which, due to mutarotation, can exist either in the α-configuration (δ_H_ = 5.18 ppm) or the β-configuration (δ_H_ = 4.58 ppm). The Ara*f* unit, which is covalently bound at position *O*3 of the internal Xyl*p* unit, shows a separated signal, which is, compared to the anomeric signals of xylose, shifted to low field (δ_H_ = 5.39 ppm). Due to the weak coupling between H1 and H2, the signal is not split into a doublet but only a (broad) singlet was detected. Complete NMR data sets were only obtained by using two-dimensional, heteronuclear NMR-experiments, such as HSQC, HSQC-TOCSY, HMBC, and H2BC experiments, which provide an extra gain in resolution by spreading the spectra in a second dimension. Therefore, the otherwise overlapping signals of the ring protons of the xylose units now appear as separate cross signals in the HSQC spectrum. Cross signals representing the positions 1 to 4 of the Ara*f* unit also facilitate peak assignment. Again, the cross signal representing the anomeric proton of α-Ara*f* is clearly isolated (δ_H/C_ = 5.39/108.22 ppm). Also, the two protons bound in C5 position of the Ara*f* unit and the Xyl*p* units, can easily be assigned, differing in their ^1^H shifts but showing the same ^13^C shift (see also **Table 2**). A comparison of the signal assignment with reference spectra of commercially available standard compounds, such as XA^3^XX, revealed significant similarities, with the exception of the additional signals belonging to the unsubstituted xylose unit.

XXA^3^X and XA^3^A^3^X were obtained in gravimetrically determined amounts of 1.3 mg and 2.9 mg, with monosaccharide compositions of xylose and arabinose in molar ratios of 78.4: 21.6 and 65.4: 34.6, respectively. Lithium adducts with *m*/*z* ratios of 685 or 817 suggest that both standard compounds have a xylose backbone consisting of four subsequent units, with one or two attached Ara*f* units, respectively. XXA^3^X showed the same HPAEC retention time as the commercially available XA^3^XX but minor differences can be observed in the NMR data: in the HSQC spectra, for example, the anomeric proton signals of the internal (Xyl*p*_I_: δ_H1/C1_ = 4.52/102.26 ppm, Xyl*p*_II_: δ_H1/C1_ = 4.47/101.94 ppm) and terminal, non-reducing Xyl*p* units (Xyl*p*_t_: δ_H1/C1_ = 4.45/102.42 ppm) showed different chemical shifts compared to XA^3^XX (Xyl*p*_I_: δ_H1/C1_ = 4.47/102.32 ppm, Xyl*p*_II_: δ_H1/C1_ = 4.51/102.31 ppm, Xyl*p*_t_: δ_H1/C1_ = 4.44/102.15 ppm). It is noteworthy that the chemical shifts of the ring protons located near the glycosidic bond between the xylose and the arabinose remain consistent, regardless of the position of the internal Xyl*p* unit to which the arabinose is attached (β-Xyl*p*_I_: δ_H2/C2_ = 3.27/73.58 ppm, δ_H3/C3_ = 3.54/74.31 ppm, δ_H4/C4_ = 3.75/76.97 ppm). The linkage position of the individual monomer units was ultimately clarified by examining the HMBC spectrum, as it shows correlations between protons and ^13^C nuclei (^2^*J*, ^3^*J* and, less commonly, ^4^*J* couplings, respectively) and thus across glycosidic bonds (^3^*J*). Consequently, the structure of XXA^3^X was unambiguously identified. However, it remains a minor by-product in GH10 and GH11 hydrolysates, as both enzymes do not usually release AXOS with its second Xyl*p* unit starting from the non-reducing end being unsubstituted (Biely et al., 2016). Since XA^3^A^3^X has an almost symmetrical structure (with the exception of the terminal xylose units being reducing or non-reducing) and the two adjacent arabinoses do not influence each other’s chemical shifts, the cross signals of the Ara*f* units in the HSQC spectrum are almost identical. The integral ratios of the anomeric proton signals in the ^1^H spectrum, however, clearly indicate that two arabinoses must be attached to the xylose backbone. Also, the ^1^H spectrum reveals that the anomeric proton signal is split into two signals as the chemical shifts are slightly different (α-Ara*f*_O3(I)_: δ_H1_ = 5.39 ppm, α-Ara*f*_O3(II)_: δ_H1_ = 5.38 ppm).

A^3^A^3^X consists of a xylotriose backbone to which two arabinose units are attached, as indicated by the monosaccharide composition of xylose and arabinose in a molar ratio of 58.9 to 41.1. The standard compound was isolated in a gravimetrically determined amount of 4.8 mg. Depending on whether the Ara*f* is bound to the terminal Xyl*p* unit or to an internal Xyl*p* unit, the position of their anomeric proton signals in the ^1^H spectrum and in the HSQC spectrum is affected (α-Ara*f*_O3(I)_: δ_H1/C1_ = 5.39/108.26 ppm, α-Ara*f*_O3(t)_: δ_H1/C1_ = 5.32/108.78 ppm) (also see **Figure 3**, **Supplementary Figure 4**). Overall, however, the Ara*f* units are represented by quite similar cross-signals. Differently, the C3H3 and C4H4 cross signals of the internal and terminal Xyl*p* units are strongly shifted compared to the corresponding cross signals in xylotriose (β-Xyl*p*_I_: δ_H3/C3_ = 3.74/77.85 ppm, δ_H4/C4_ = 3.83/74.27 ppm; β-Xyl*p*_t_: δ_H3/C3_ = 3.57/82.12 ppm, δ_H4/C4_ = 3.66/68.43 ppm). Remarkably, the NMR data also shows great similarity to those of other AXOS. Both the HSQC cross signals of the substituted terminal and internal Xyl*p* and the bound Ara*f* exhibit the same chemical shifts as in A^3^X and XA^3^X, respectively.

AXs in wheat contain a significant portion of Xyl*p* units, which are disubstituted at positions *O*2 and *O*3 (Izydorczyk and Biliaderis, 1993; Schooneveld-Bergmans et al., 1999). Consequently, it is not surprising that XA^2+3^XX is also among the main products in GH10 and GH11 hydrolysates, with recovered quantities of 2.9 mg. Differently, A^2+3^XX is only found in GH10 hydrolysates because GH11 xylanases typically do not release AXOS containing a substituted non-reducing end (Biely et al., 2016; Capetti et al., 2021). Only minor differences can be observed in the HSQC cross signals of the Ara*f* units compared to monosubstituted AXOS, such as XA^2^XX and XA^3^XX, except for the anomeric signals (α-Ara*f*_O2(II)_: δ_H1/C1_ = 5.22/109.35 ppm, α-Ara*f*_O3(II)_: δ_H1/C1_ = 5.27/108.72 ppm). However, the cross signals of the disubstituted Xyl*p* unit that represent the linkage positions *O*2 and *O*3 are specific to this structural element (β-Xyl*p*_II_: δ_H2/C2_ = 3.57/79.22 ppm, β-Xyl*p*_II_: δ_H3/C3_ = 3.82/78.18 ppm). Also, *O*2 substitution of the internal Xyl*p* unit significantly affects the chemical shift of the anomeric proton signal. Thus, the C1H1 signal (β-Xyl*p*_II_: δ_H1/C1_ = 4.63/100.49 ppm) is clearly isolated from those of unsubstituted (β-Xyl*p*_II_: δ_H1/C1_ = 4.46/102.36 ppm) and *O*3-monosubstituted (β-Xyl*p*_II_: δ_H1/C1_ = 4.46/102.26 ppm) Xyl*p* units. However, it is close to the signal of *O*2-monosubstituted Xyl*p* units (β-Xyl*p*_II_: δ_H1/C1_ = 4.58/100.69 ppm).

**Figure 4:**
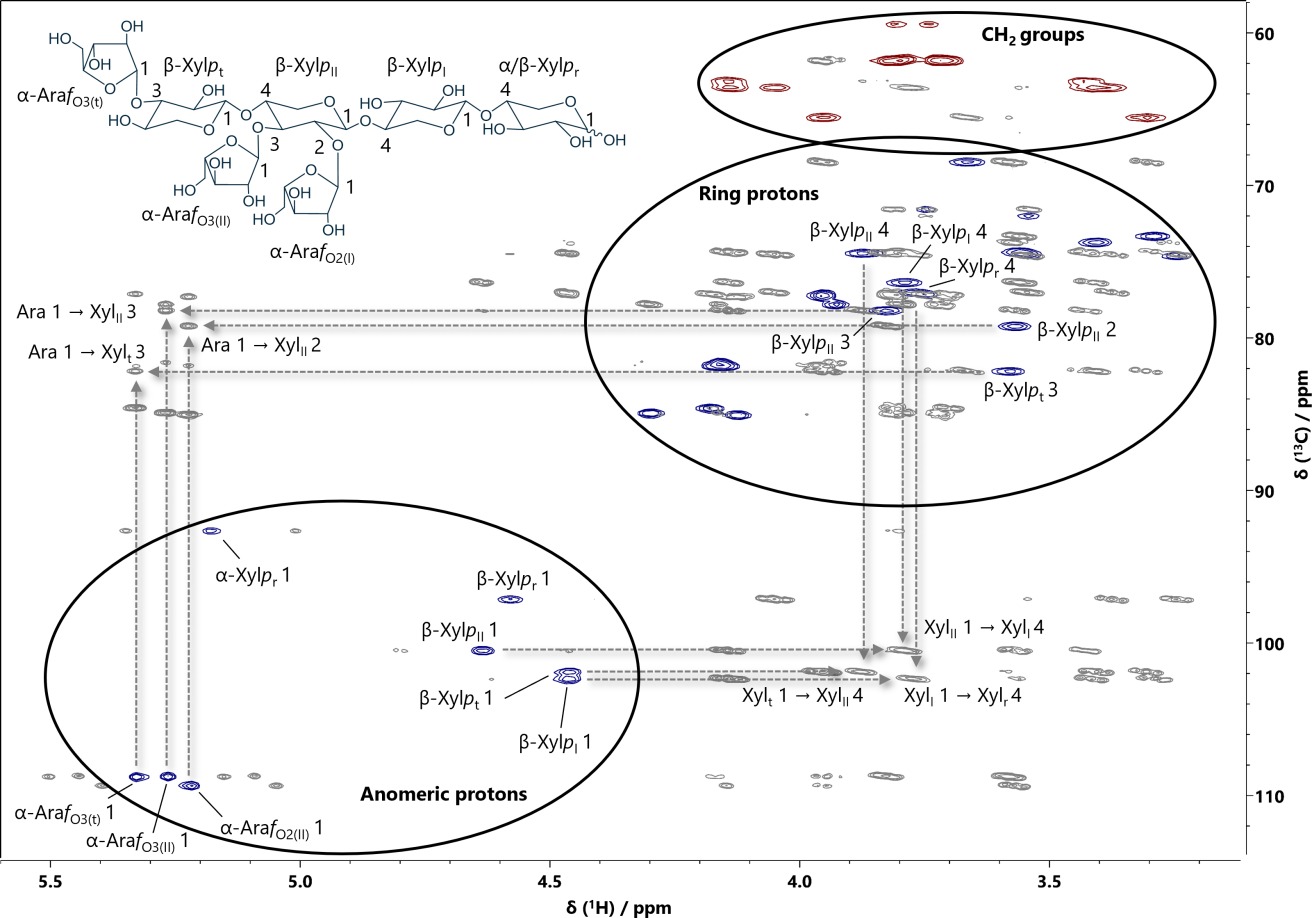
Superimposed HSQC and HMBC spectra of A^3^A^2+3^XX (structure shown). The spectra were recorded on a 500 MHz spectrometer and calibrated against the chemical shift of acetone (δ_H/C_ = 2.22/30.89 ppm). The positions involved in the glycosidic bonds have been marked accordingly. HSQC signals are shown either in blue (CH groups) or in red (CH_2_ groups). Grey colored HMBC signals result from ^2^*J*, ^3^*J* or ^4^*J* couplings between protons and ^13^C nuclei and were used to determine the linkage positions of the monomers. Abbreviations correspond to those shown in **Figure 2**.

A^3^A^2+3^XX was identified as a predominant component in the GH10 hydrolysate, with gravimetric amounts reaching up to 8.3 mg total across multiple BioGel fractions. In the HPAEC-PAD/MS analysis, a double lithium adduct with *m*/*z* = 478 was detected, corresponding to a heptamer. The ^1^H NMR spectrum (**Figure 3**) reveals that the anomeric proton signals of the bound Ara*f* exhibit similar chemical shifts as those observed in A^3^X, A^3^A^3^X and XA^2+3^XX, respectively. **Figure 4** shows the superimposed HSQC and HMBC spectra of A^3^A^2+3^XX. The multiplicity-edited HSQC experiment helps to differentiate between CH_2_ groups of the *O*5 position of pentoses and the CH groups of the remaining ring protons. Just as the anomeric protons, the CH_2_ groups of both the Xyl*p* and Ara*f* units are clearly separated from the remaining ring protons because they have smaller ^13^C chemical shifts (δ_C_ = 59–66 ppm). Again, the cross signals in the HMBC spectrum were used to identify the positions of the glycosidic linkages, for example the intense signals at δ_H/C_ = 3.88/101.87 ppm (Xyl*p*_t_ 1 → Xyl*p*_II_ 4), δ_H/C_ = 3.80/100.45 ppm (Xyl*p*_II_ 1 → Xyl*p*_I_ 4) and δ_H/C_ = 3.76/102.38 ppm (Xyl*p*_I_ 1 → Xyl*p*_r_ 4) result from the β-(1→4)-glycosidic bonds between the Xyl*p* units in the backbone of A^3^A^2+3^XX. It has also been confirmed that the α-configurated Ara*f* units are bound to the *O*3 position of the terminal Xyl*p* unit and the *O*2+*O*3 positions of the second internal Xyl*p* unit via their lactol group (δ_H/C_ = 5.33/82.16 ppm (Ara*f* 1 → Xyl*p*_t_ 3), δ_H/C_ = 5.27/78.19 ppm (Ara*f* 1 → Xyl*p*_II_ 3) and δ_H/C_ = 5.22/79.21 ppm (Ara*f* 1 → Xyl*p*_II_ 2).

The corresponding oligosaccharide XA^3^A^2+3^XX was isolated and purified from the GH11 hydrolysate with gravimetric amounts of 5.8 mg. The monosaccharide composition (xylose: 61.8 mol%, arabinose: 39.2 mol%) and the double lithium adduct with *m*/*z* = 544 indicate a xylopentose with three attached arabinose units. Again, the NMR data are very similar to that of the respective smaller AXOS, such as XA^3^X and XA^2+3^XX, which contain the same structural elements (see **Table 2**).

A gravimetrically determined amount of 4.7 mg total of A^2+3^A^2+3^XX with two adjacent disubstituted Xyl*p* units was isolated from the GH10 hydrolysate. A balanced ratio of arabinose to xylose in the monosaccharide composition (xylose: 52.3 mol%, arabinose: 47.7 mol%) and a double lithium adduct with *m*/*z* = 544 already suggested a highly substituted AXOS. It is evident from the anomeric signals of the Ara*f* units in the ^1^H NMR spectrum (see **Figure 3**) that the arabinose units influence each other regarding their chemical shifts (δ_H_ = 5.20 - 5.30 ppm) as they are different to those observed in smaller AXOS that contain the same structural elements, for example A^2+3^XX and XA^2+3^XX. Accordingly, cross signals of the terminal, disubstituted Xyl*p* unit (β-Xyl*p*_t_: δ_H3/C3_ = 3.66/83.13 ppm) and the inner disubstituted Xyl*p* unit (β-Xyl*p*_II_: δ_H2/C2_ = 3.56/79.46 ppm, δ_H3/C3_ = 3.85/78.05 ppm) also exhibit slightly different shifts compared to A^2+3^XX (β-Xyl*p*_t_: δ_H3/C3_ = 3.68/82.82 ppm) and XA^2+3^XX (β-Xyl*p*_II_: δ_H2/C2_ = 3.59/79.46 ppm, δ_H3/C3_ = 3.82/78.25 ppm). Therefore, the NMR data of A^2+3^A^2+3^XX show that two consecutive disubstituted Xyl*p* units, albeit to a small extent, produce different signals than disubstituted Xyl*p* units that are isolated from each other by sections of unsubstituted Xyl*p* units. As demonstrated by A^3^A^2+3^XX and XA^3^A^2+3^XX above, an *O*3-monosubstituted Xyl*p* unit located in the vicinity of the disubstituted Xyl*p* unit likewise exerts no influence on its signal position. However, since no AXOS containing an *O*2-monosubstituted Xyl*p* unit adjacent to the disubstituted Xyl*p* unit were isolated, its influence on the signal position cannot be assessed. This must be taken into account when considering these signals as potential marker signals for this specific structural element. AXOS with two consecutive disubstituted Xyl*p* units have already been detected in the enzymatic digestion of wheat AX (Gruppen et al., 1992b).

A^2+3^XA^2+3^XX was the largest AXOS that was isolated and purified from the enzymatic digestion of wheat AX but only in a quantity of less than 1 mg. The molar ratio of xylose and arabinose was 54.4 to 45.6, and a double lithium adduct with *m*/*z* = 611 was detected, thus representing an oligosaccharide with nine pentoses. As with the other AXOS, mass spectral data was obtained from HPAEC-PAD/MS analysis; however, this was difficult to detect because the suppressor was not able to reduce the salt load properly due to the high amounts of salt in the eluent (i.e., large amounts of acetate at the end of the gradient program that are not affected by the suppressor), which increased the noise in the mass spectrum. It is notable that the HSQC cross signals are comparable to those of the smaller AXOS such as A^2+3^XX and XA^2+3^XX due to the presence of an additional unsubstituted Xyl*p* unit separating the disubstituted Xyl*p* units. In contrast to A^2+3^A^2+3^XX, there is no mutual influence between the disubstituted Xyl*p* units and the associated arabinoses on each other’s chemical shifts. Interestingly, the signals from the two *O*2-bound arabinose units were indistinguishable, that is, their chemical shifts were identical. However, integrating the signal volumes revealed that these signals are the result of two structural elements: the signal volumes of the *O*2-bound arabinose units were approximately twice that of the (distinguishable) *O*3-bound arabinose units, indicating that two CH groups from different arabinose units contributed to these signals.

## 4 Discussion

Nine AXOS standard compounds were isolated and purified from the GH10 and GH11 hydrolysate of the alkaline extracted wheat flour AX. Despite the fact that wheat flour AX had a notably low degree of substitution, the isolated structures demonstrated that wheat AX contain more heavily substituted regions. These regions can be hydrolyzed, at least to a certain extent, by *endo*-xylanases of GH families 10 and 11. Both xylanases can attack higher substituted regions of wheat AX but require at least two consecutive unsubstituted Xyl*p* units in order to cleave the xylan backbone. Therefore, areas of AX that are too densely substituted are not broken down by *endo*-xylanases from GH families 10 and 11. However, GH10 *endo*-xylanases are less hampered by xylan substitution than their GH11 counterparts (Pollet et al., 2010; Biely et al., 2016). Structural models on wheat AX propose that the distribution of Ara*f* along the xylan backbone is nonrandom and that highly substituted regions contain high proportions of disubstituted units along with sections of consecutive monosubstituted xylose units (Gruppen et al., 1992a, 1993). Dervilly-Pinel et al. (2004) applied a statistical model to assess the random distribution of differently substituted xylose residues and compared the results with experimental data from well-characterized wheat endosperm water-extractable AXs. Their analysis revealed significant deviations between the experimental values and the model’s predictions. Notably, the frequency of disubstitution was substantially higher than what would be expected under a random distribution. This is consistent with the structures that have been isolated in this work, most notably A^2+3^A^2+3^XX. A lot of research has been done on the isolation of AXOS standard compounds from cereal sources following enzymatic hydrolysis (Gruppen et al., 1992b; Verbruggen et al., 1998; McCleary et al., 2015). Thus, it is not surprising that no entirely novel structural element of wheat AX is presented here. It should be noted that some liberated oligo- and/or polysaccharides of the GH10 and GH11 hydrolysates (corresponding to the first BioGel fractions, **Figure 1**) were too large (degree of polymerization > 9 pentose units) to allow for further chromatographic purification; thus, they were not included in this study. Nonetheless, it is assumed that these compounds show structural similarities to those presented. For example, Gruppen et al. (1992b) described an AXOS with ten pentose units, closely resembling A^2+3^XA^2+3^XX but with an additional Xyl*p* unit in the backbone. However, we were able to obtain full NMR data sets even for the most complex structural units (A^2+3^XA^2+3^XX, A^2+3^A^2+3^XX, XA^3^A^2+3^XX, A^3^A^2+3^XX) as we succeeded in the isolation of sufficient amounts of these AXOS in sufficient purity. Certainly, also the use of a cryoprobe in combination with the application of non-uniform sampling allowed for a full structural characterization and recording of complete ^1^H and ^13^C NMR datasets. In order to use this publication as a comprehensive tool in the identification of (A)XOS we also give ^1^H and ^13^C NMR datasets for commercially available XOS and AXOS in the Supplementary Material (**Supplementary Table 2**). The provided NMR data can, for instance, serve as a reference for highlighting differences in the substitution patterns of AX, not only between different genera (e.g., *Triticum* vs. *Secale*) or species (e.g., *Triticum aestivum* vs. *Triticum* sp*elta*), but also between different varieties or cultivars. In addition, the possible effects of cultivation methods and environmental conditions on the AX structures of cereal grains can be investigated. Furthermore, structural changes of AX as a consequence of food processing steps can be monitored at different stages. For example, during malt preparation in the brewing process, soluble AX from malted barley grains are degraded by endogenous enzymes lowering the overall molecular weight of AX (Michiels et al., 2024). However, the effect of enzyme activities on the fine structure of AX, particularly regarding their substitution patterns, remains poorly understood at this stage. Similar effects are observed when *endo*-xylanases are added during dough preparation, i.e., when water-insoluble AX is broken down into smaller, water-soluble fragments that can have a positive effect on the rheological properties of bread (Guo et al., 2018). Detailed 2D-NMR structural analyses of these fragments will enhance our understanding of the liberated oligosaccharides and may result in new hypotheses how these fragments may interact with gluten network.

In this context, it is important to mention that the evaluation of all NMR data clearly showed that AXOS that contain the same structural elements produce the same signals, regardless of their degree of polymerization. This allows us to identify specific marker signals that are indicative of a particular structural element in the overall AX structure. Two-dimensional NMR profiling methods for the semiquantitative determination of specific structural elements of cell wall polysaccharides were already described (Wefers and Bunzel, 2016; Schendel and Bunzel, 2022) and a comparable approach will be developed by using the presented data.

## Data Availability

The original contributions presented in the study are included in the article/**Supplementary Material**. Further inquiries can be directed to the corresponding author/s.
