# Supplementary material for: 2D-NMR characterization of higher substituted oligosaccharides isolated from enzymatic wheat flour arabinoxylan hydrolysates
Source: Front Plant Sci. 2026 Mar 9;17:1784230. doi: 10.3389/fpls.2026.1784230 (PMC13006628; doi:10.3389/fpls.2026.1784230)
Supplement: Supplementary file 1 [file DataSheet1.pdf]

2D-NMR characterization of higher substituted oligosaccharides isolated from enzymatic wheat flour arabinoxylan hydrolysates

Lukas Sitter, Mirko Bunzel

Supplementary Material

Supplementary Table 1: Purification steps for the isolated arabinoxylooligosaccharides (AXOS) from the enzymatic hydrolysates of the wheat flour arabinoxylan after BioGel-P2 separation. The nomenclature of the AXOS corresponds to the naming system suggested by Faure *et al*., 2009. GH, glycoside hydrolase family

AXOS	BioGel-P2 fraction		Chromatographic purification steps			HPAEC-PAD purity
	GH10 hydrolysis	GH11 hydrolysis	HILIC	PGC	HPAEC	
XA^3X	5	-	-	X	-	> 95 %
XXA^3X	4	-	X	X	-	> 90 %
A^3A^3X	4	-	X	X	-	> 95 %
$XA^{2+3}XX$	3,4	-	-	X	X	> 95 %
XA^3A^3X	3	-	-	X	-	> 90 %
$A^3A^{2+3}XX$	2,3	-	X	X	-	> 95 %
$XA^3A^{2+3}XX$	-	3	X	X	-	> 90 %
$A^{2+3}A^{2+3}XX$	2	-	X	X	-	> 95 %
$A^{2+3}XA^{2+3}XX$	2	-	X	X	X	> 95 %

HSQC spectra of isolated arabinoxylooligosaccharide standard compounds

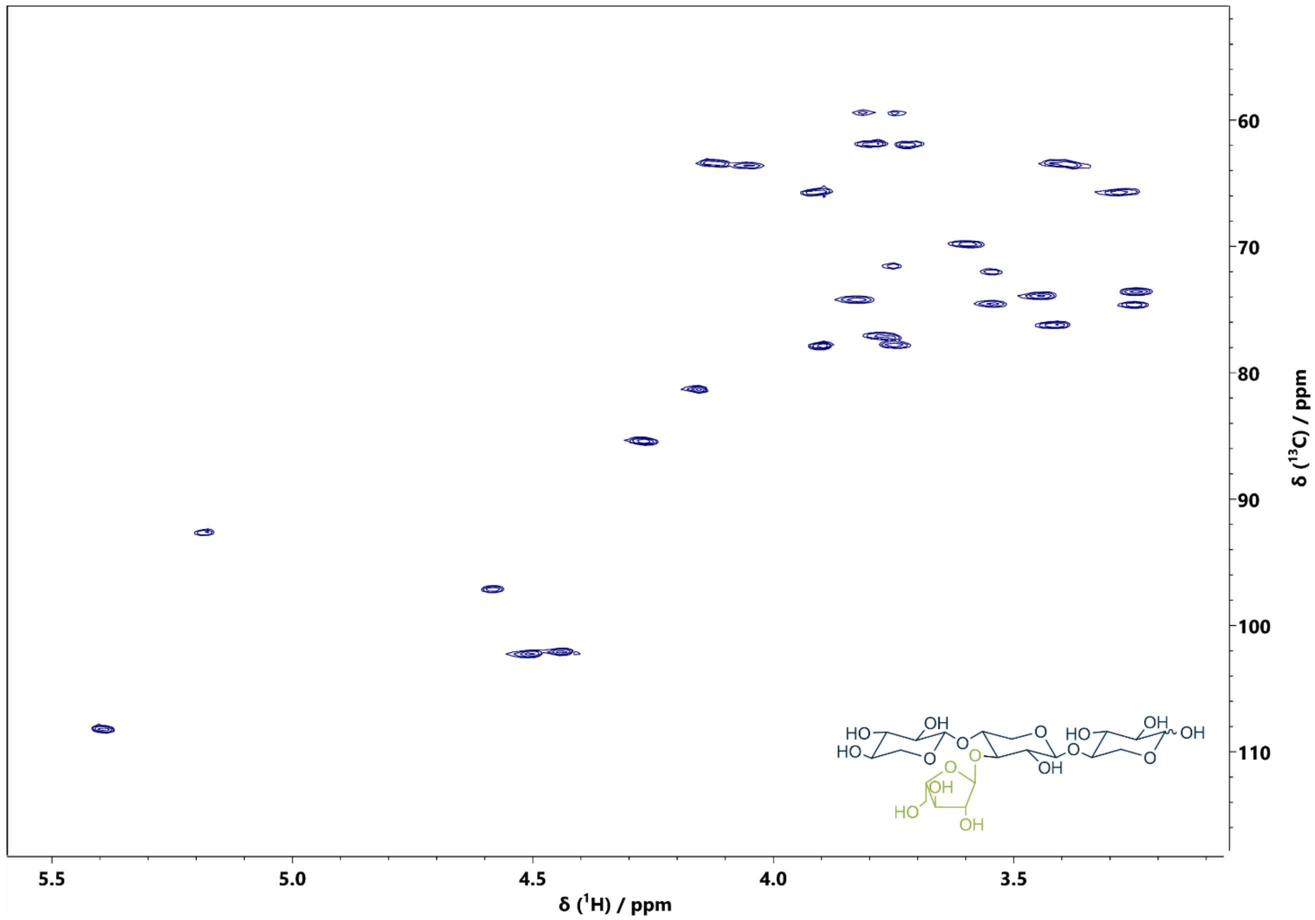

Supplementary Figure 1: HSQC spectrum and chemical structure of the arabinoxylooligosaccharide XA^3X. The acquistion was performed in D_2O using a 500 MHz spectrometer and chemical shifts were referenced against acetone ($\delta_{H/C}$ = 2.22/30.89 ppm). The color scheme of the chemical structure corresponds to **Figure 2**.

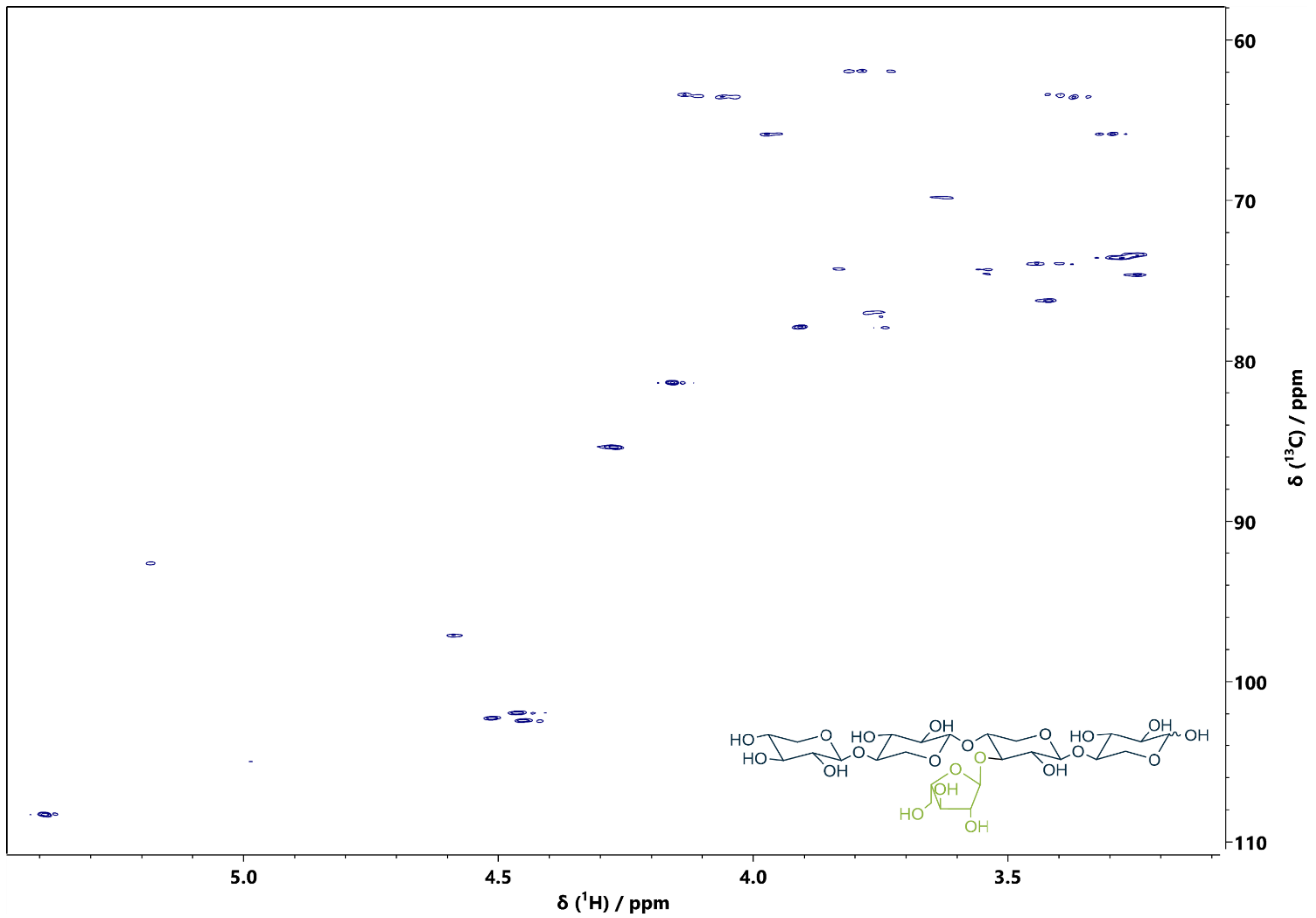

Supplementary Figure 2: HSQC spectrum and chemical structure of the arabinoxylooligosaccharide XXA^{3}X. The acquistion was performed in D_2O using a 500 MHz spectrometer and chemical shifts were referenced against acetone ($\delta_{H/C}$ = 2.22/30.89 ppm). The color scheme of the chemical structure corresponds to **Figure 2**.

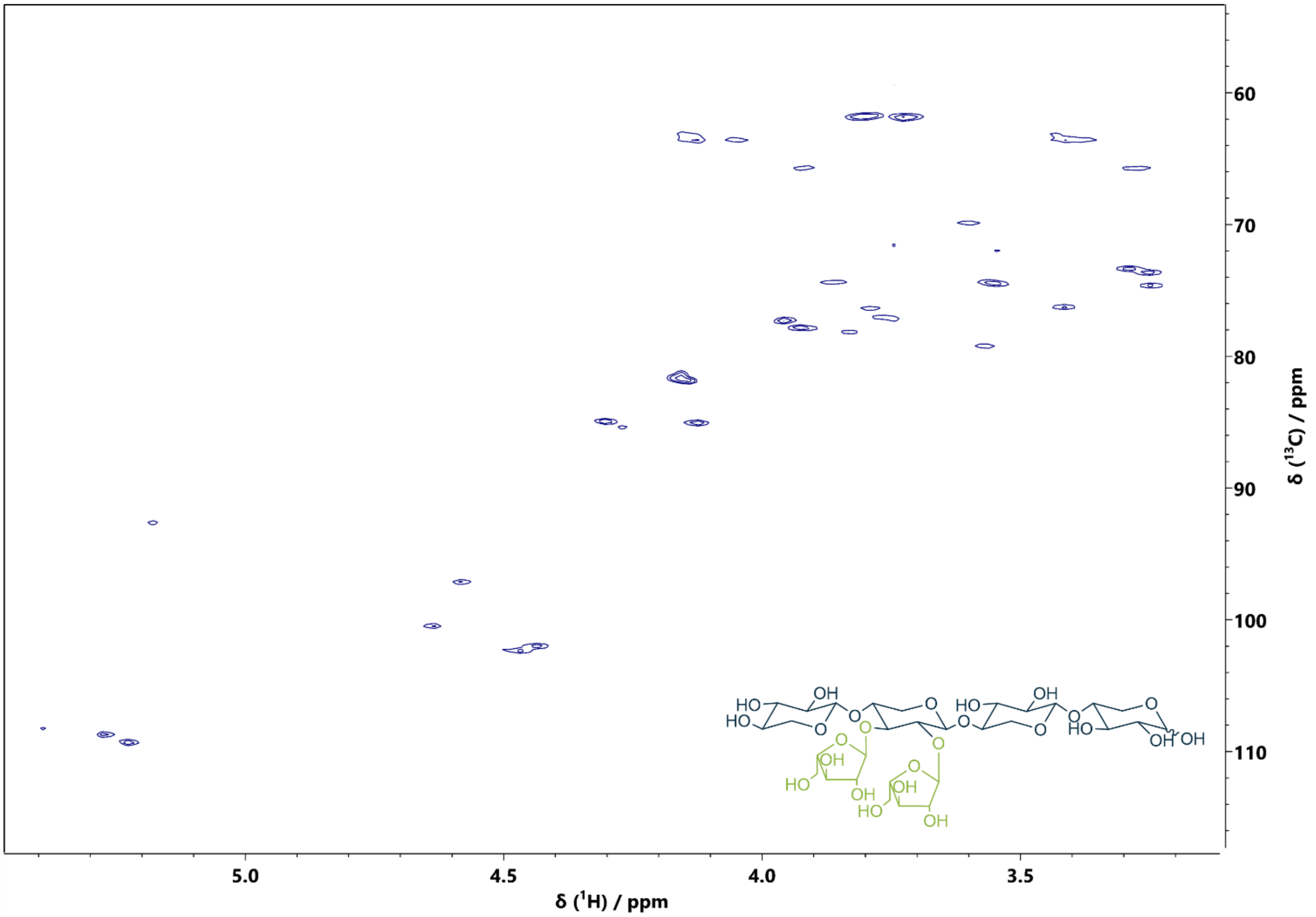

Supplementary Figure 3: HSQC spectrum and chemical structure of the arabinoxylooligosaccharide $XA^{2+3}XX$. The acquistion was performed in D_2O using a 500 MHz spectrometer and chemical shifts were referenced against acetone ($\delta_{H/C}$ = 2.22/30.89 ppm). The color scheme of the chemical structure corresponds to **Figure 2**.

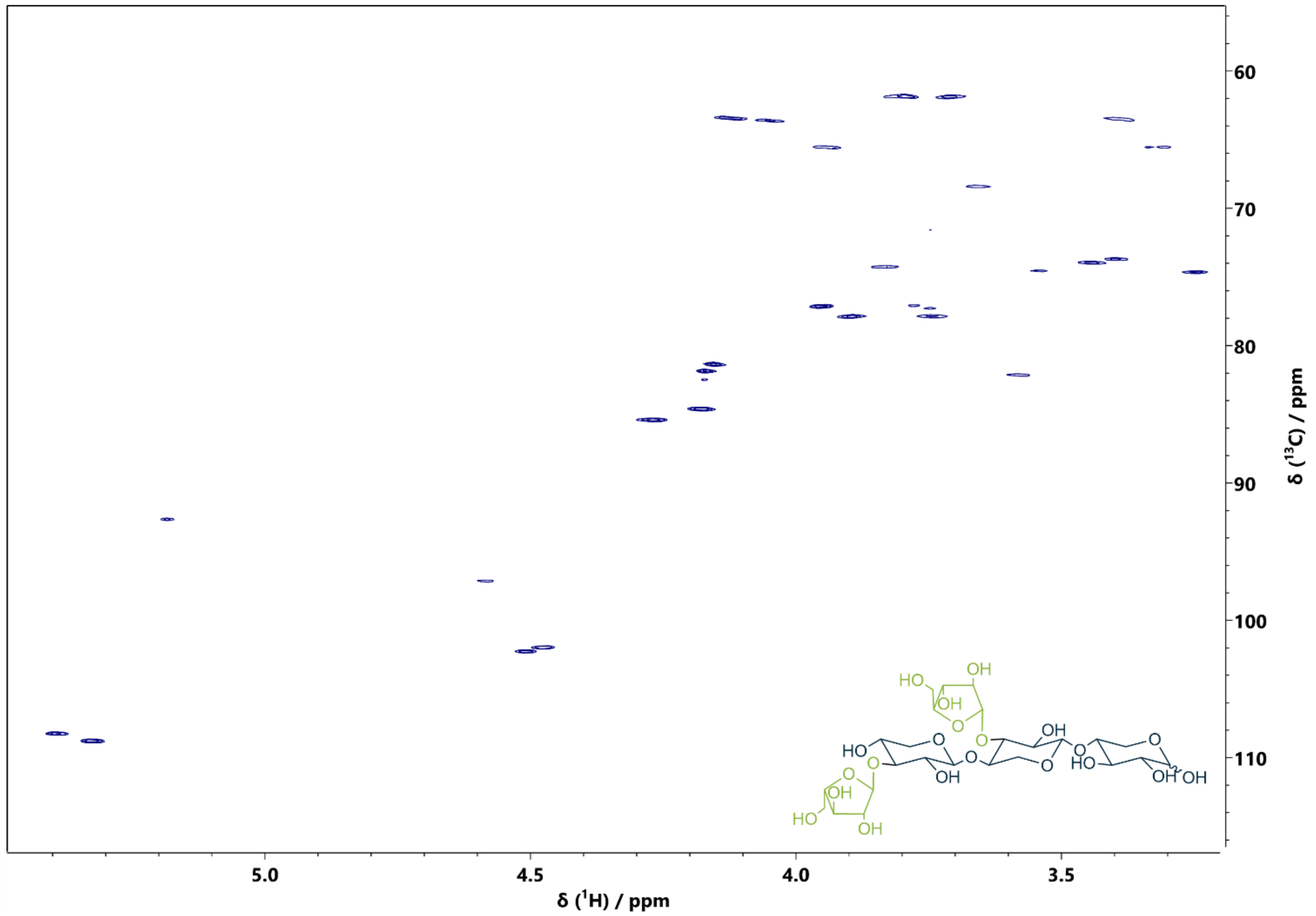

Supplementary Figure 4: HSQC spectrum and chemical structure of the arabinoxylooligosaccharide A^3A^3X. The acquistion was performed in D_2O using a 500 MHz spectrometer and chemical shifts were referenced against acetone ($\delta_{H/C}$ = 2.22/30.89 ppm). The color scheme of the chemical structure corresponds to **Figure 2**.

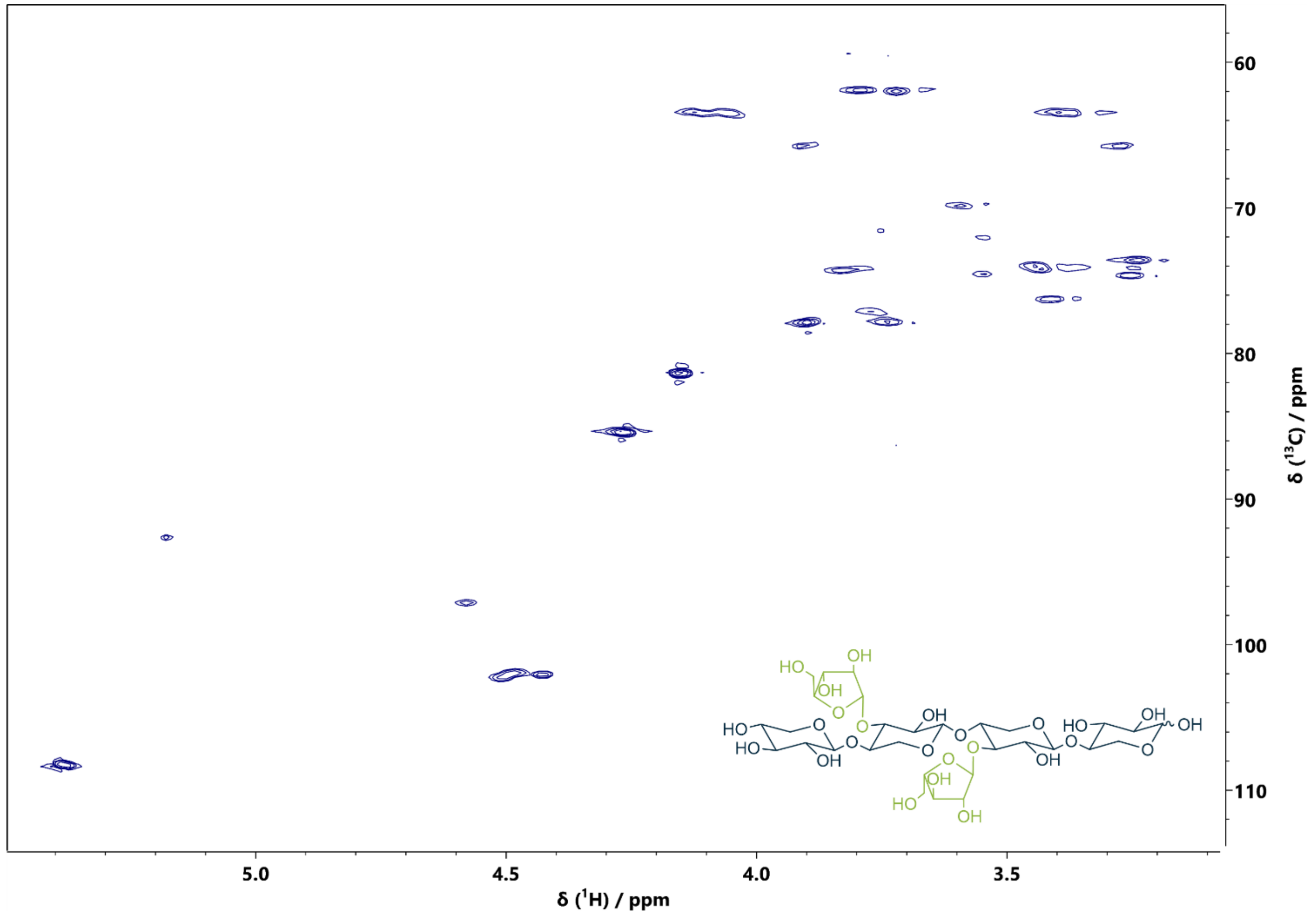

Supplementary Figure 5: HSQC spectrum and chemical structure of the arabinoxylooligosaccharide XA^3A^3X. The acquistion was performed in D_2O using a 500 MHz spectrometer and chemical shifts were referenced against acetone ($\delta_{H/C}$ = 2.22/30.89 ppm). The color scheme of the chemical structure corresponds to **Figure 2**.

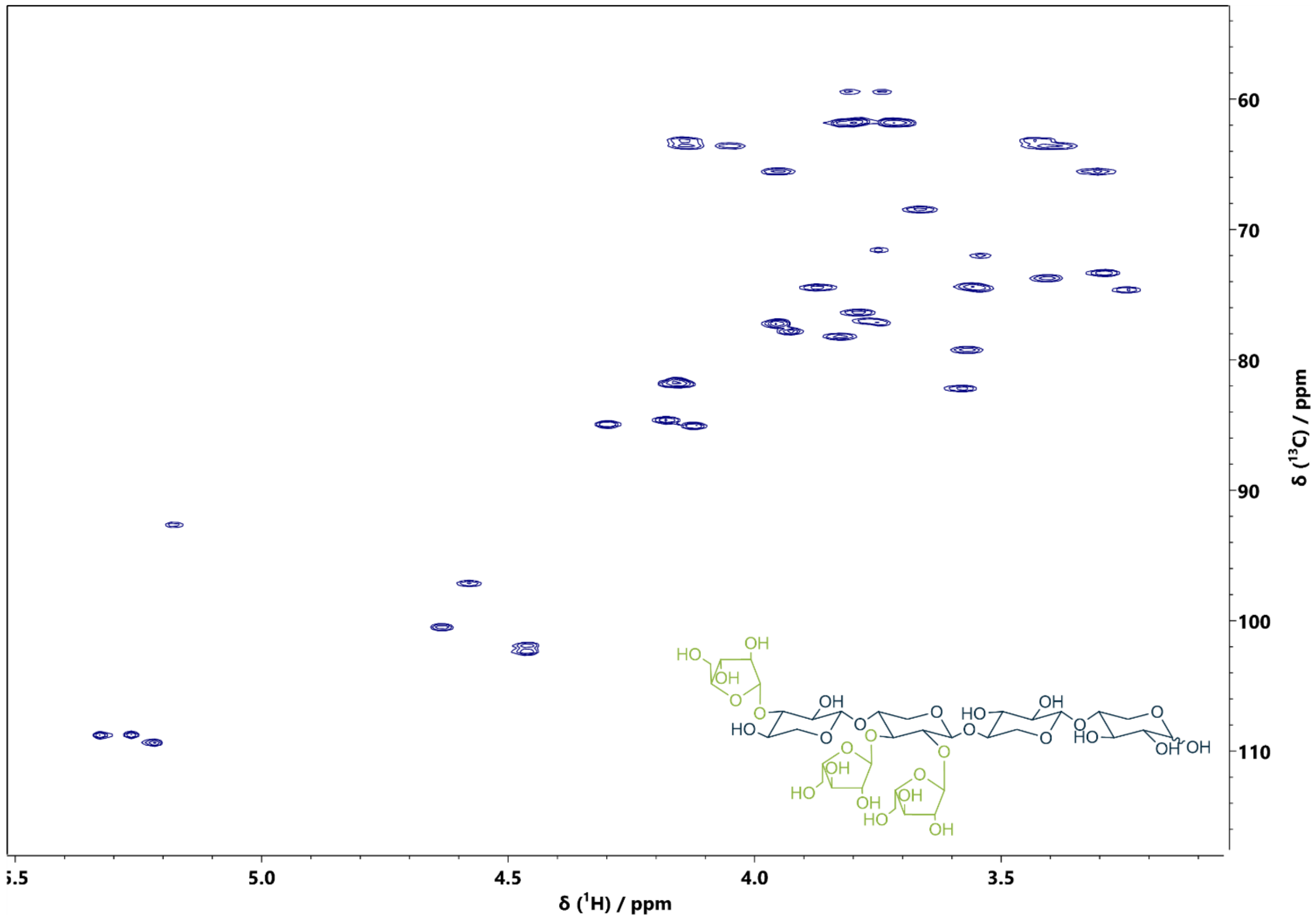

Supplementary Figure 6: HSQC spectrum and chemical structure of the arabinoxylooligosaccharide $A^3A^{2+3}XX$. The acquistion was performed in D_2O using a 500 MHz spectrometer and chemical shifts were referenced against acetone ($\delta_{H/C}$ = 2.22/30.89 ppm). The color scheme of the chemical structure corresponds to **Figure 2**.

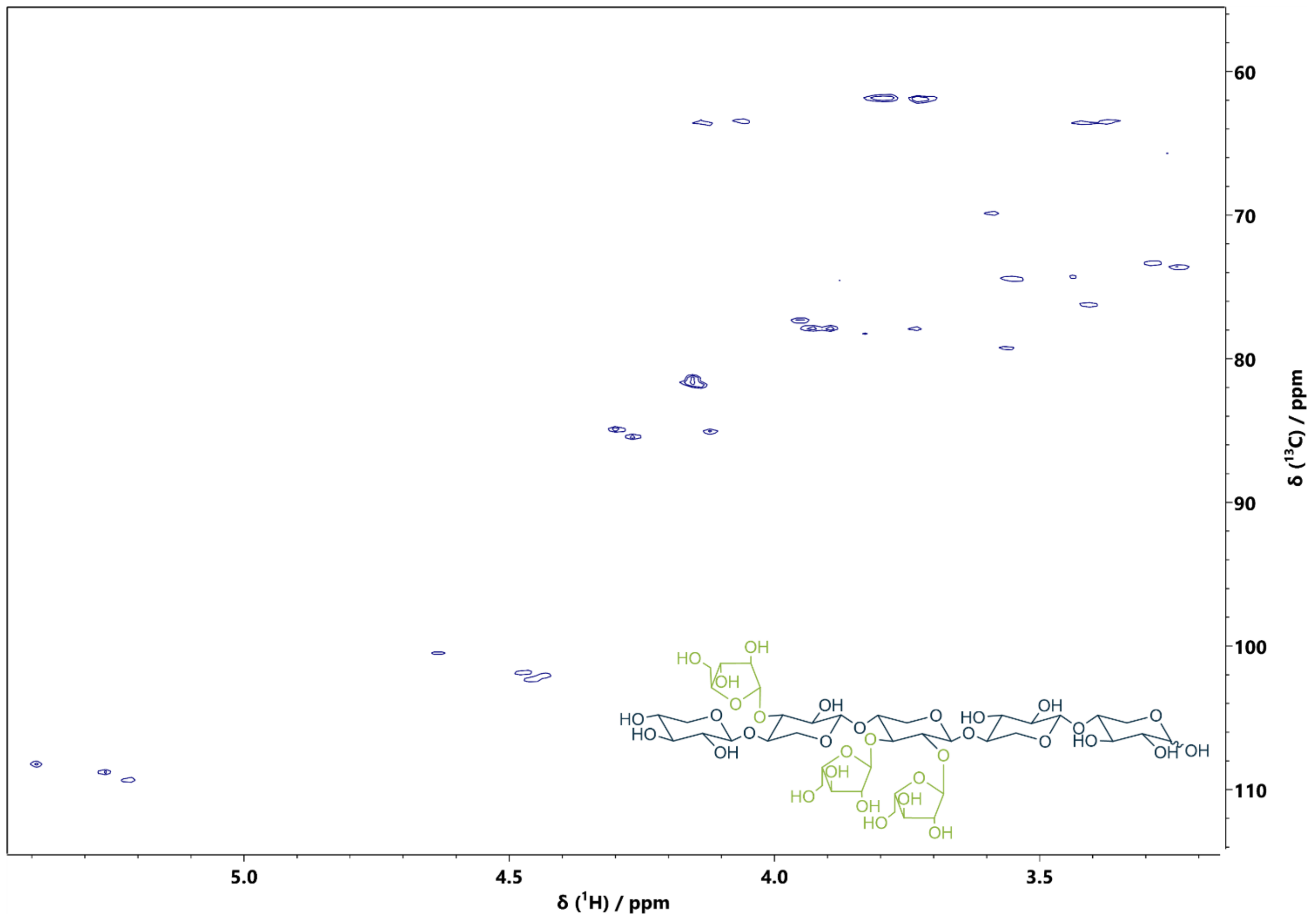

Supplementary Figure 7: HSQC spectrum and chemical structure of the arabinoxylooligosaccharide $XA^3A^{2+3}XX$. The acquistion was performed in D_2O using a 500 MHz spectrometer and chemical shifts were referenced against acetone ($\delta_{H/C}$ = 2.22/30.89 ppm). The color scheme of the chemical structure corresponds to **Figure 2**.

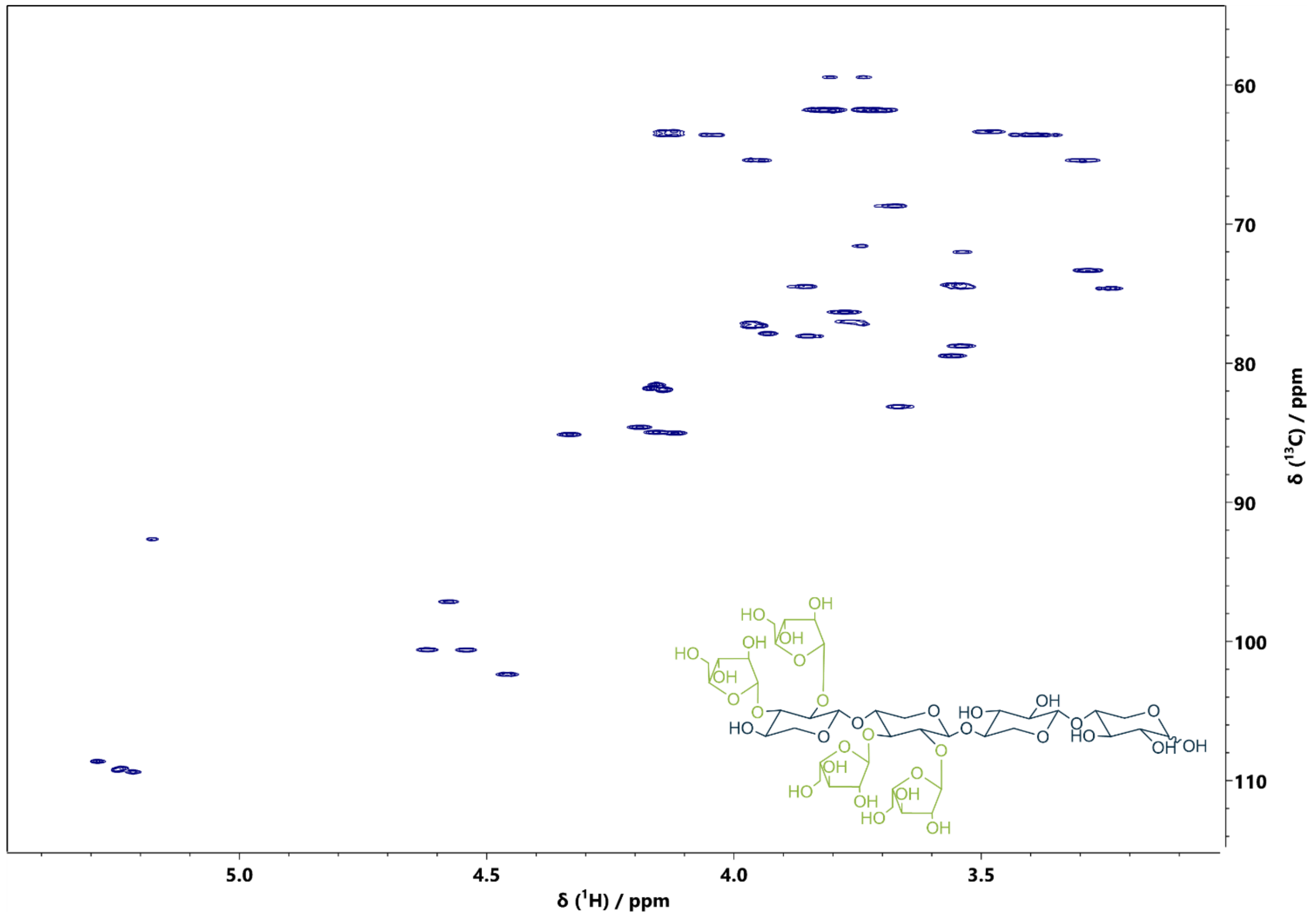

Supplementary Figure 8: HSQC spectrum and chemical structure of the arabinoxylooligosaccharide $A^{2+3}A^{2+3}XX$. The acquistion was performed in D_2O using a 500 MHz spectrometer and chemical shifts were referenced against acetone ($\delta_{H/C}$ = 2.22/30.89 ppm). The color scheme of the chemical structure corresponds to **Figure 2**.

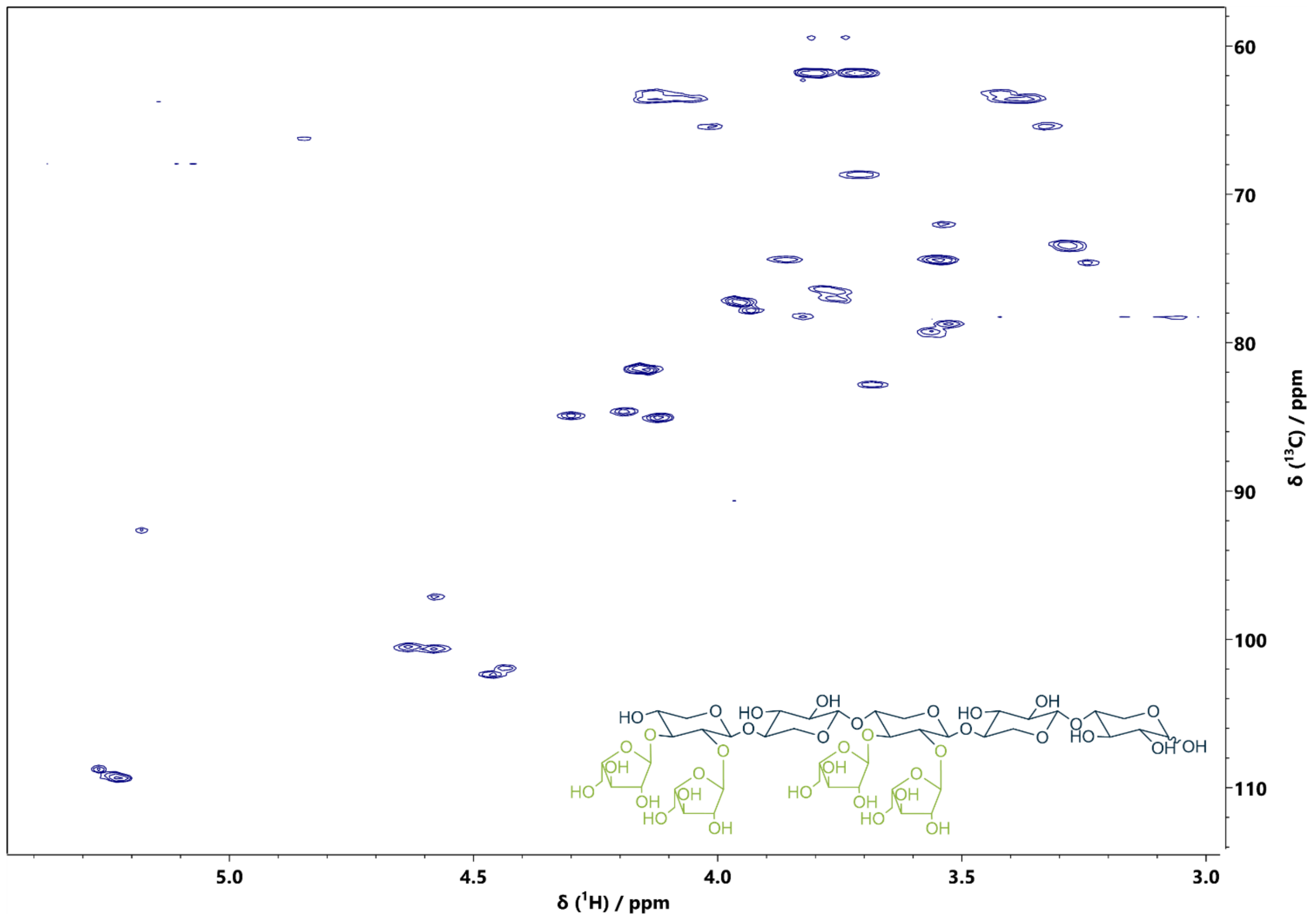

Supplementary Figure 9: HSQC spectrum and chemical structure of the arabinoxylooligosaccharide $A^{2+3}XA^{2+3}XX$. The acquistion was performed in D_2O using a 500 MHz spectrometer and chemical shifts were referenced against acetone ($\delta_{H/C}$ = 2.22/30.89 ppm). The color scheme of the chemical structure corresponds to **Figure 2**.

NMR data and HSQC spectra of commercially available xylo- and arabinoxylooligosaccharide standard compounds

Supplementary Table 2: ^{1}H and ^{13}C chemical shifts of the commercially available standard compounds of xylooligosaccharides (XOS) and arabinoxylooligosaccharides (AXOS). The vicinal couplings constants ($^{3}J_{H,H}$) to the neighboring proton is given in Hz in parentheses. The nomenclature of the XOS and AXOS is taken from the naming system according to Fauré *et al.* (2009). Abbreviations correspond to those shown in **Figure 2**.

Unit	H1	H2	H3	H4	H5
	C1	C2	C3	C4	C5
			**X_2**		
**α-Xyl*p*$_r$**	5.18 (3.65)	3.53	3.75	3.74	3.80/3.74
	92.69	72.06	71.58	77.24	59.52
**β-Xyl*p*$_r$**	4.58 (7.85)	3.24	3.54	3.77	4.05/3.37
	97.14	74.65	74.57	77.08	63.65
**β-Xyl*p*$_t$**	4.45 (7.98)	3.24	3.42	3.62	3.96/3.30
	102.52	73.44	76.27	69.88	65.83
			**X_3**		
**α-Xyl*p*$_r$**	5.18 (3.65)	3.53	3.75	3.74	3.80/3.74
	92.69	72.06	71.58	77.24	59.44
**β-Xyl*p*$_r$**	4.58 (7.90)	3.24	3.54	3.77	4.05/3.37
	97.14	74.65	74.57	76.99	63.65
**β-Xyl*p*$_I$**	4.47 (7.91)	3.28	3.55	3.78	4.10/3.37
	102.30	73.36	74.32	77.00	63.65
**β-Xyl*p*$_t$**	4.45 (7.82)	3.25	3.42	3.62	3.96/3.30
	102.48	73.44	76.27	69.88	65.87
			**X_4**		
**α-Xyl*p*$_r$**	5.18 (3.65)	3.53	3.75	3.74	3.80/3.74
	92.69	72.06	71.58	77.24	59.44
**β-Xyl*p*$_r$**	4.58 (7.90)	3.24	3.54	3.78	4.05/3.37
	97.14	74.65	74.57	76.99	63.65
**β-Xyl*p*$_{I-II}$**	4.47	3.29	3.55	3.79	4.10/3.37
	102.32	73.36	74.33	76.99	63.64
**β-Xyl*p*$_t$**	4.45 (7.88)	3.25	3.42	3.62	3.96/3.30
	102.48	73.44	76.27	69.88	65.87
			**X_5**		
**α-Xyl*p*$_r$**	5.18 (3.66)	3.54	3.74	3.74	3.81/3.74
	92.69	72.06	71.58	77.24	59.44
**β-Xyl*p*$_r$**	4.58 (7.90)	3.24	3.54	3.78	4.04/3.37
	97.14	74.65	74.57	76.99	63.65
**β-Xyl*p*$_{I-III}$**	4.47	3.28	3.55	3.79	4.09/3.37
	102.32	73.36	74.33	77.00	63.65

**β-Xyl*p*t**	4.45 (7.90)	3.25	3.42	3.62	3.96/3.30
	102.49	73.44	76.27	69.87	65.90
**X_6**					
**α-Xyl*p*r**	5.18 (3.65)	3.54	3.74	3.74	3.81/3.74
	92.69	72.06	71.58	77.24	59.45
**β-Xyl*p*r**	4.58 (7.87)	3.24	3.54	3.77	4.05/3.37
	97.14	74.65	74.57	77.00	63.65
**β-Xyl*p*I-IV**	4.47	3.29	3.55	3.79	4.10/3.37
	102.32	73.36	74.33	77.00	63.65
**β-Xyl*p*t**	4.45 (7.99)	3.25	3.42	3.62	3.96/3.30
	102.48	73.44	76.27	69.88	65.87
**A^3X**					
**α-Xyl*p*r**	5.18 (3.65)	3.53	3.74	3.75	3.81/3.74
	92.69	72.06	71.58	77.24	59.53
**β-Xyl*p*r**	4.58 (7.85)	3.25	3.54	3.77	4.05/3.37
	97.14	74.65	74.57	77.16	63.65
**β-Xyl*p*t**	4.48 (7.88)	3.40	3.59	3.69	4.00/3.33
	102.40	73.52	82.09	68.42	65.67
**α-Ara*f*O3**	5.33	4.17	3.95	4.18	3.81/3.70
	108.79	81.85	77.16	84.68	61.87
**A^2XX**					
**α-Xyl*p*r**	5.18 (3.66)	3.54	3.74	3.74	3.80/3.74
	92.69	72.06	71.58	77.16	59.44
**β-Xyl*p*r**	4.58 (7.93)	3.24	3.54	3.76	4.05/3.37
	97.14	74.65	74.57	77.08	63.65
**β-Xyl*p*I**	4.46 (7.77)	3.28	3.55	3.78	4.13/3.40
	102.40	73.36	74.41	76.75	63.73
**β-Xyl*p*t**	4.55 (7.51)	3.41	3.55	3.66	3.98/3.30
	100.94	78.78	76.34	69.72	65.67
**α-Ara*f*O2**	5.27	4.16	3.94	4.14	3.81/3.71
	109.11	81.69	77.32	85.25	61.95
**$A^{2+3}XX$**					
**α-Xyl*p*r**	5.17 (3.66)	3.54	3.74	3.74	3.81/3.74
	92.64	72.01	71.57	77.21	59.45
**β-Xyl*p*r**	4.58 (7.78)	3.24	3.54	3.77	4.04/3.37
	97.15	74.63	74.55	77.01	63.59
**β-Xyl*p*I**	4.46 (7.82)	3.28	3.55	3.78	4.14/3.41
	102.40	73.36	74.41	76.59	63.65
**β-Xyl*p*t**	4.59 (7.49)	3.53	3.69	3.71	4.01/3.34
	100.65	78.74	82.81	68.67	65.41
**α-Ara*f*O2**	5.23	4.14	3.95	4.12	3.82/3.72
	109.35	81.84	77.34	85.02	61.86
**α-Ara*f*O3**	5.24	4.17	3.97	4.19	3.80/3.71
	109.11	81.76	77.13	84.66	61.82

XA²XX + XA³XX mixture					
**α-Xyl*p*r**	5.18 (3.60)	3.74	3.53	3.74	3.81/3.74
	92.69	71.58	72.06	77.24	59.45
**β-Xyl*p*r**	4.58 (7.85)	3.24	3.54	3.78	4.05/3.36
	97.14	74.65	74.57	77.06	63.65
**β-Xyl*p*I (XA²XX)**	4.46	3.29	3.55	3.78	4.13/3.40
	102.34	73.36	74.33	76.67	63.70
**β-Xyl*p*I (XA³XX)**	4.47	3.29	3.55	n. d.	4.10/3.37
	102.32	73.36	74.33	n. d.	63.65
**β-Xyl*p*II (XA²XX)**	4.58	3.45	3.69	n. d.	4.11/3.39
	100.70	78.61	74.33	n. d.	63.45
**β-Xyl*p*II (XA³XX)**	4.51 (7.79)	3.44	3.74	3.82	4.11/3.39
	102.31	73.96	77.81	74.25	63.45
**β-Xyl*p*t (XA²XX)**	4.46	3.25	3.43	3.63	3.96/3.30
	102.56	73.44	76.27	69.88	65.87
**β-Xyl*p*t (XA³XX)**	4.44	3.24	3.41	3.60	3.91/3.27
	102.15	73.60	76.27	69.89	65.75
**α-Ara*f*O2(II)**	5.28	4.16	3.94	4.13	4.12/3.39
	109.11	81.69	77.32	85.25	63.46
**α-Ara*f*O3(II)**	5.39	4.15	3.90	4.27	4.12/3.39
	108.22	81.36	77.89	85.41	63.46

n. d. = not determinable

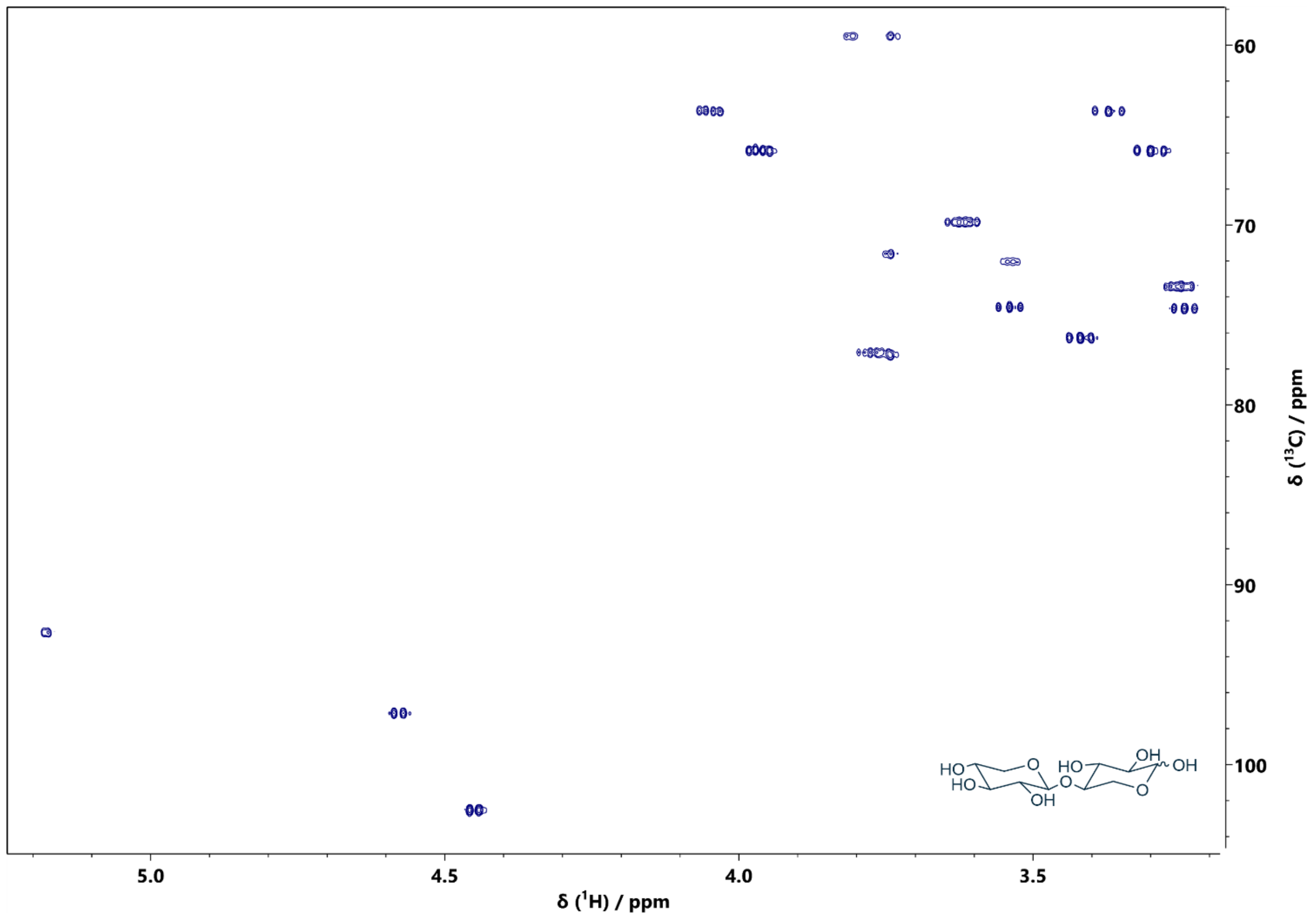

Supplementary Figure 10: HSQC spectrum and chemical structure of the xylooligosaccharide X_2. The acquistion was performed in D_2O using a 500 MHz spectrometer and chemical shifts were referenced against acetone ($\delta_{H/C}$ = 2.22/30.89 ppm). The color scheme of the chemical structure corresponds to **Figure 2**.

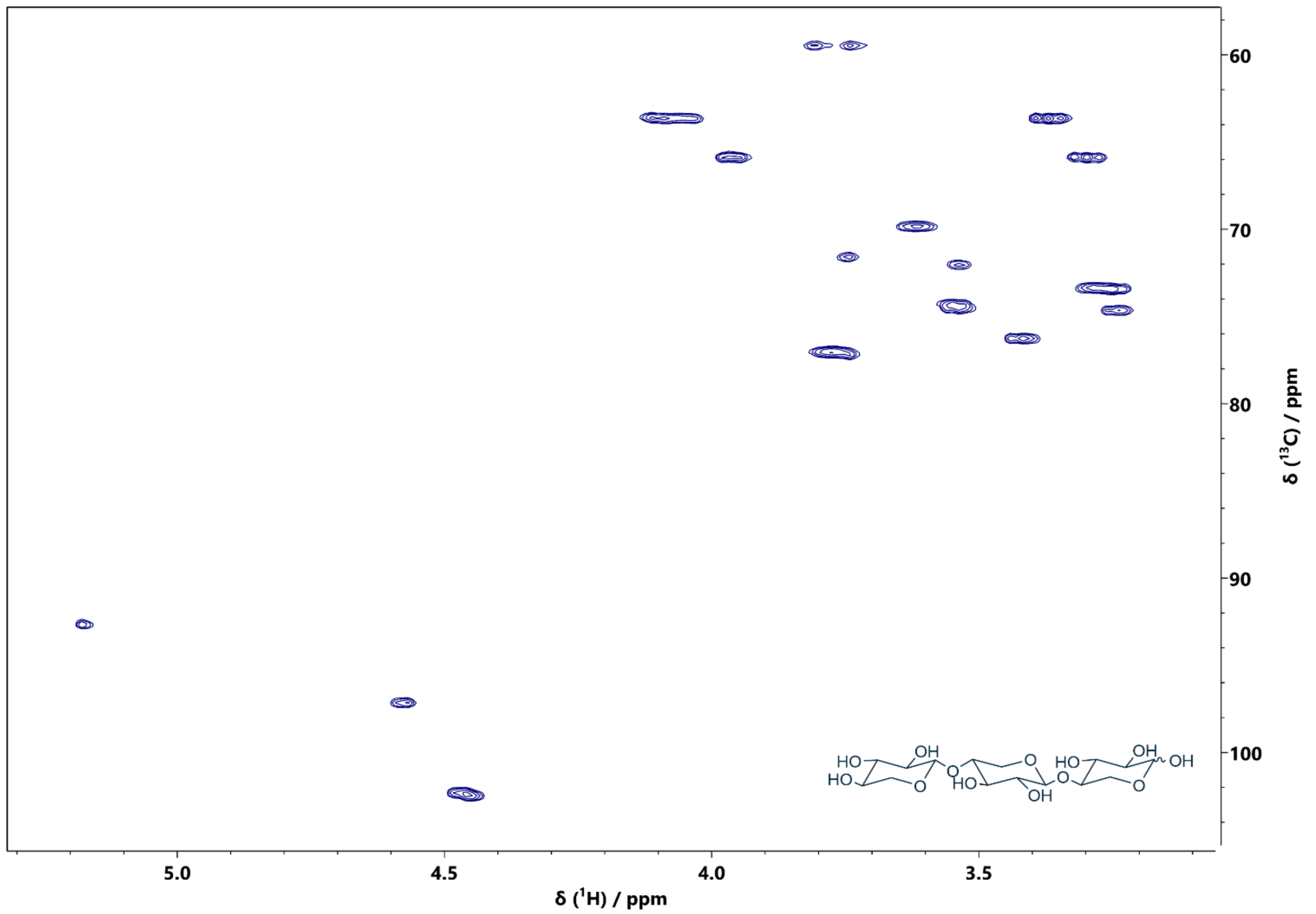

Supplementary Figure 11: HSQC spectrum and chemical structure of the xylooligosaccharide X_3. The acquistion was performed in D_2O using a 500 MHz spectrometer and chemical shifts were referenced against acetone ($\delta_{H/C}$ = 2.22/30.89 ppm). The color scheme of the chemical structure corresponds to **Figure 2**.

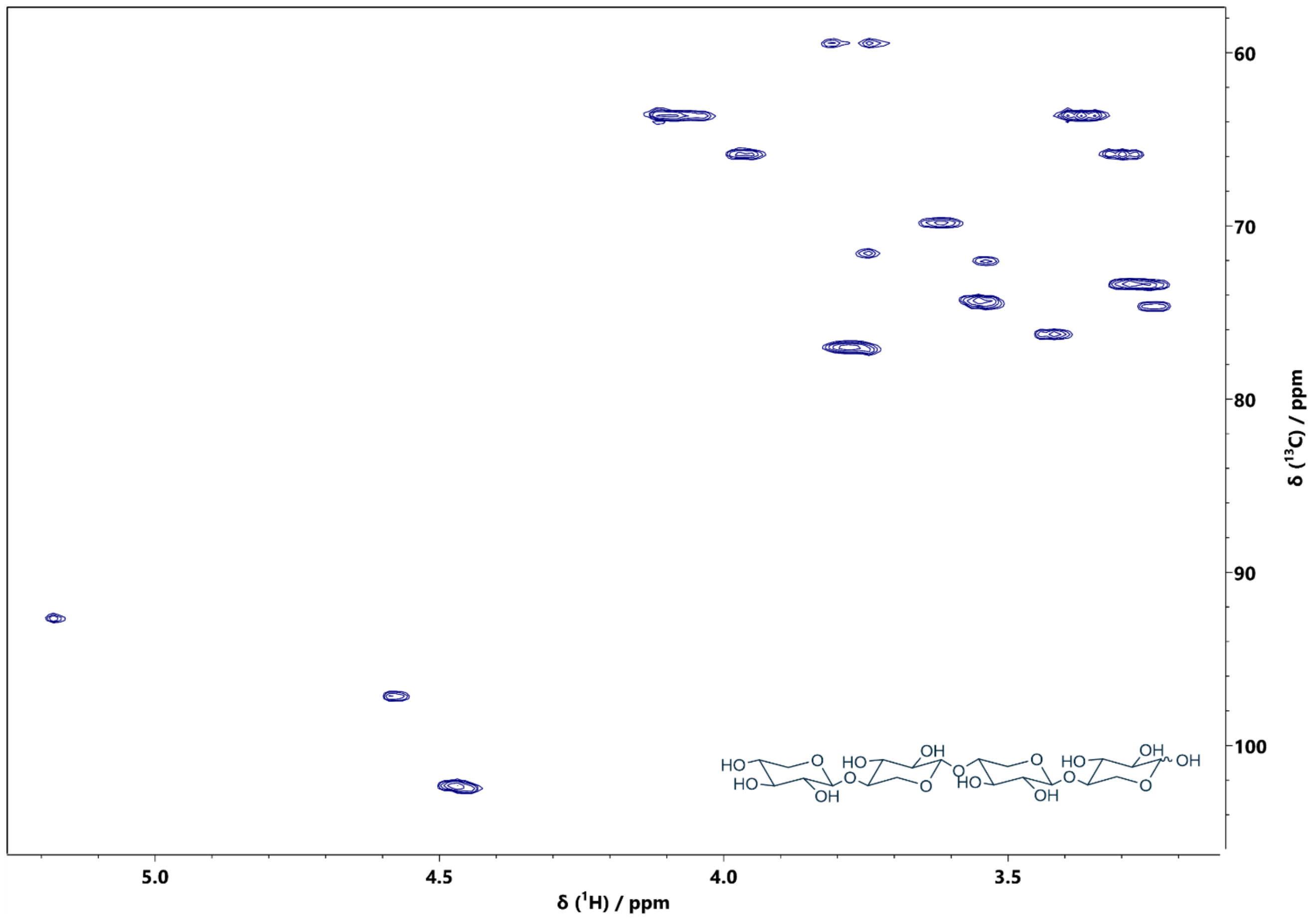

Supplementary Figure 12: HSQC spectrum and chemical structure of the xylooligosaccharide X_4. The acquistion was performed in D_2O using a 500 MHz spectrometer and chemical shifts were referenced against acetone ($\delta_{H/C}$ = 2.22/30.89 ppm). The color scheme of the chemical structure corresponds to **Figure 2**.

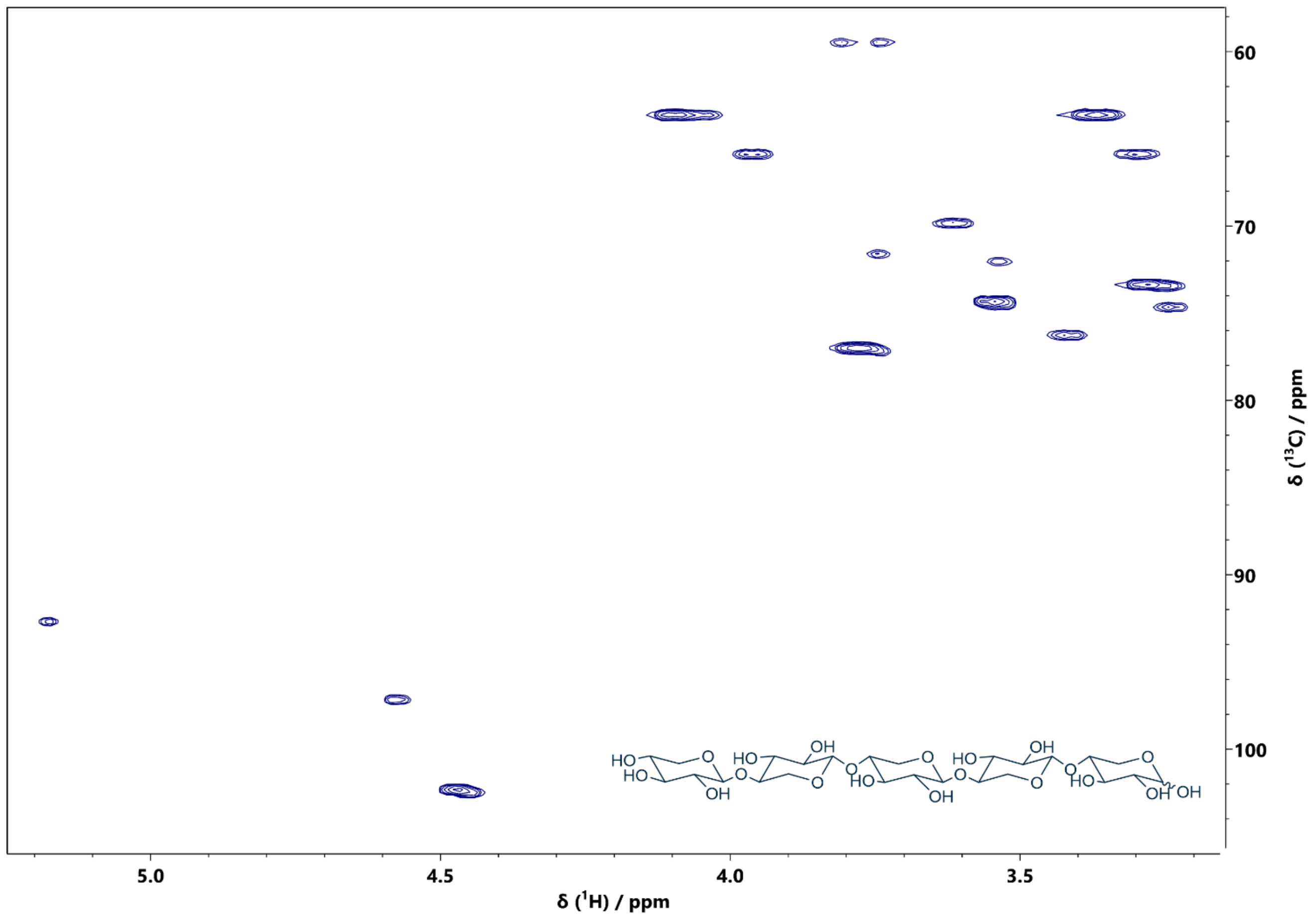

Supplementary Figure 13: HSQC spectrum and chemical structure of the xylooligosaccharide X_5. The acquistion was performed in D_2O using a 500 MHz spectrometer and chemical shifts were referenced against acetone ($\delta_{H/C}$ = 2.22/30.89 ppm). The color scheme of the chemical structure corresponds to **Figure 2**.

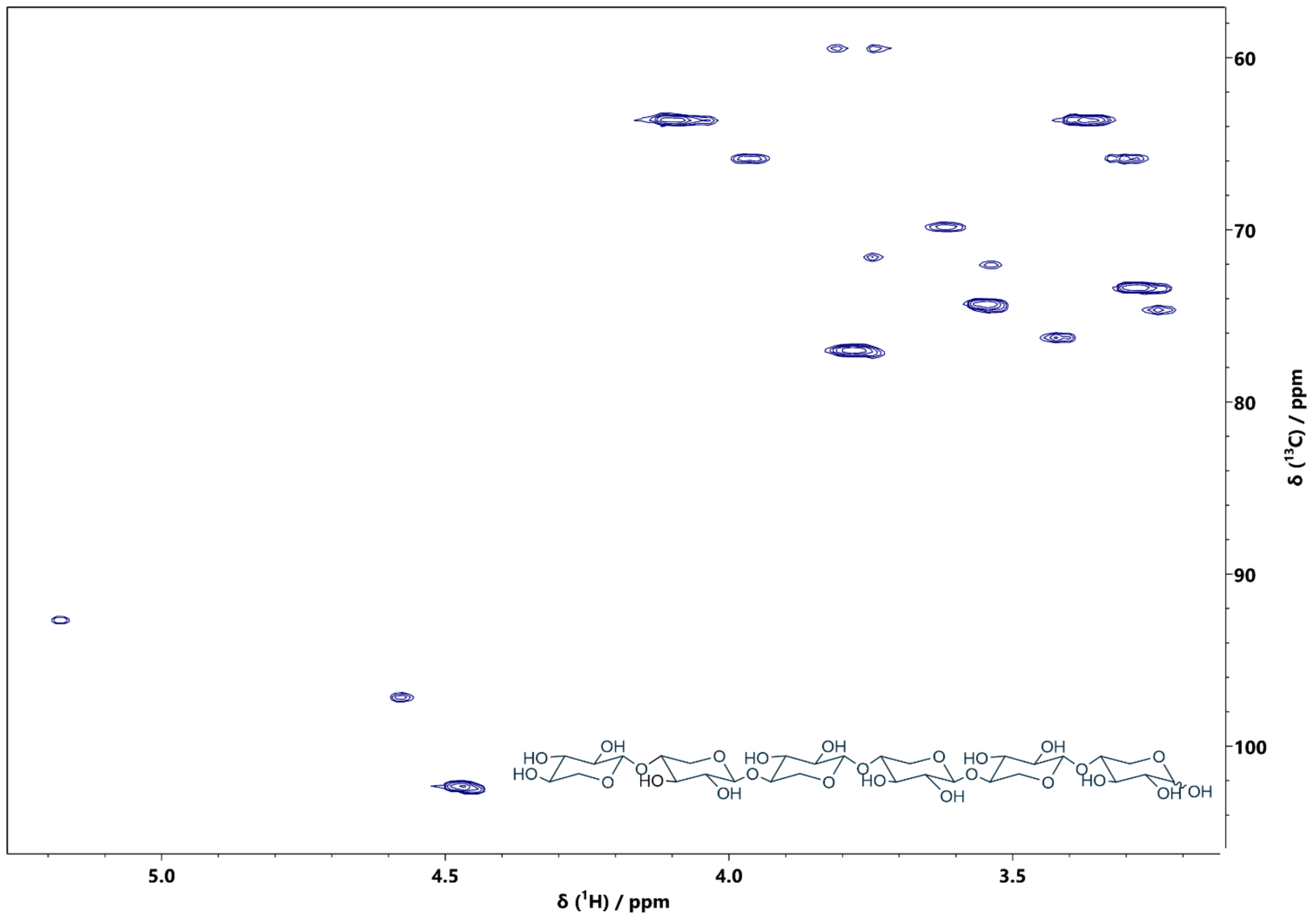

Supplementary Figure 14: HSQC spectrum and chemical structure of the xylooligosaccharide X_6. The acquistion was performed in D_2O using a 500 MHz spectrometer and chemical shifts were referenced against acetone ($\delta_{H/C}$ = 2.22/30.89 ppm). The color scheme of the chemical structure corresponds to **Figure 2**.

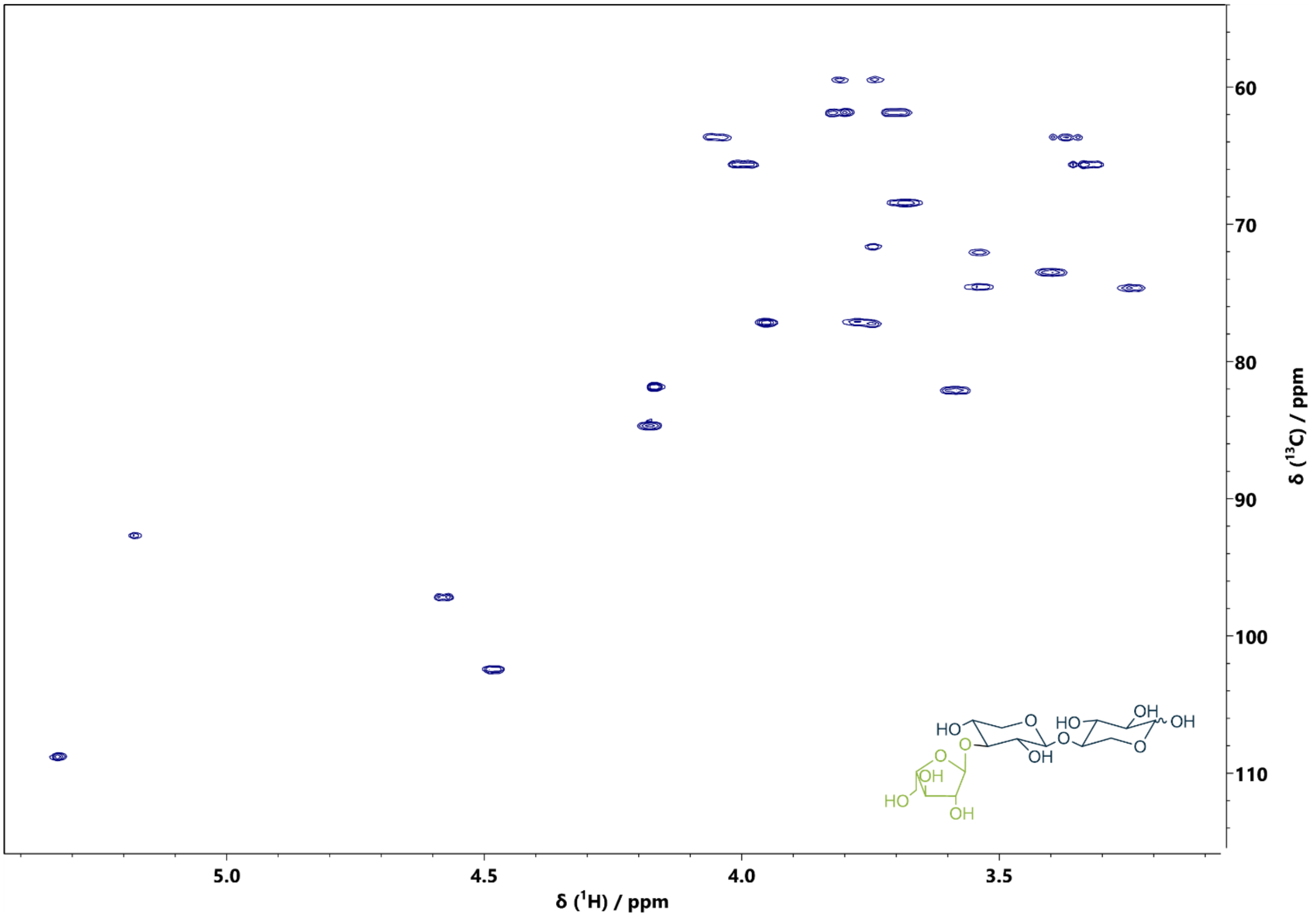

Supplementary Figure 15: HSQC spectrum and chemical structure of the arabinoxylooligosaccharide A^3X. The acquistion was performed in D_2O using a 500 MHz spectrometer and chemical shifts were referenced against acetone ($\delta_{H/C}$ = 2.22/30.89 ppm). The color scheme of the chemical structure corresponds to **Figure 2**.

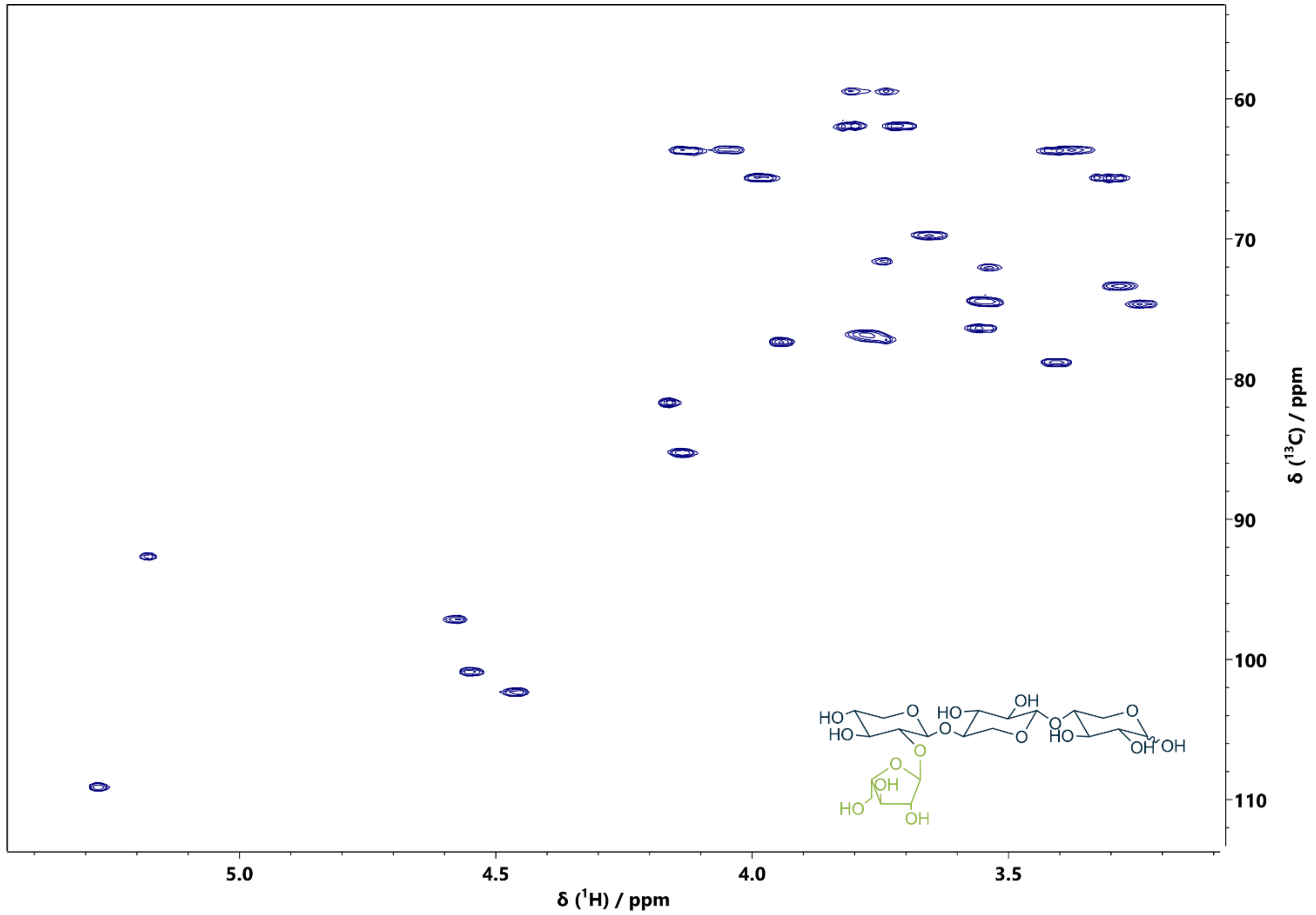

Supplementary Figure 16: HSQC spectrum and chemical structure of the arabinoxylooligosaccharide A^2XX. The acquistion was performed in D_2O using a 500 MHz spectrometer and chemical shifts were referenced against acetone ($\delta_{H/C}$ = 2.22/30.89 ppm). The color scheme of the chemical structure corresponds to **Figure 2**.

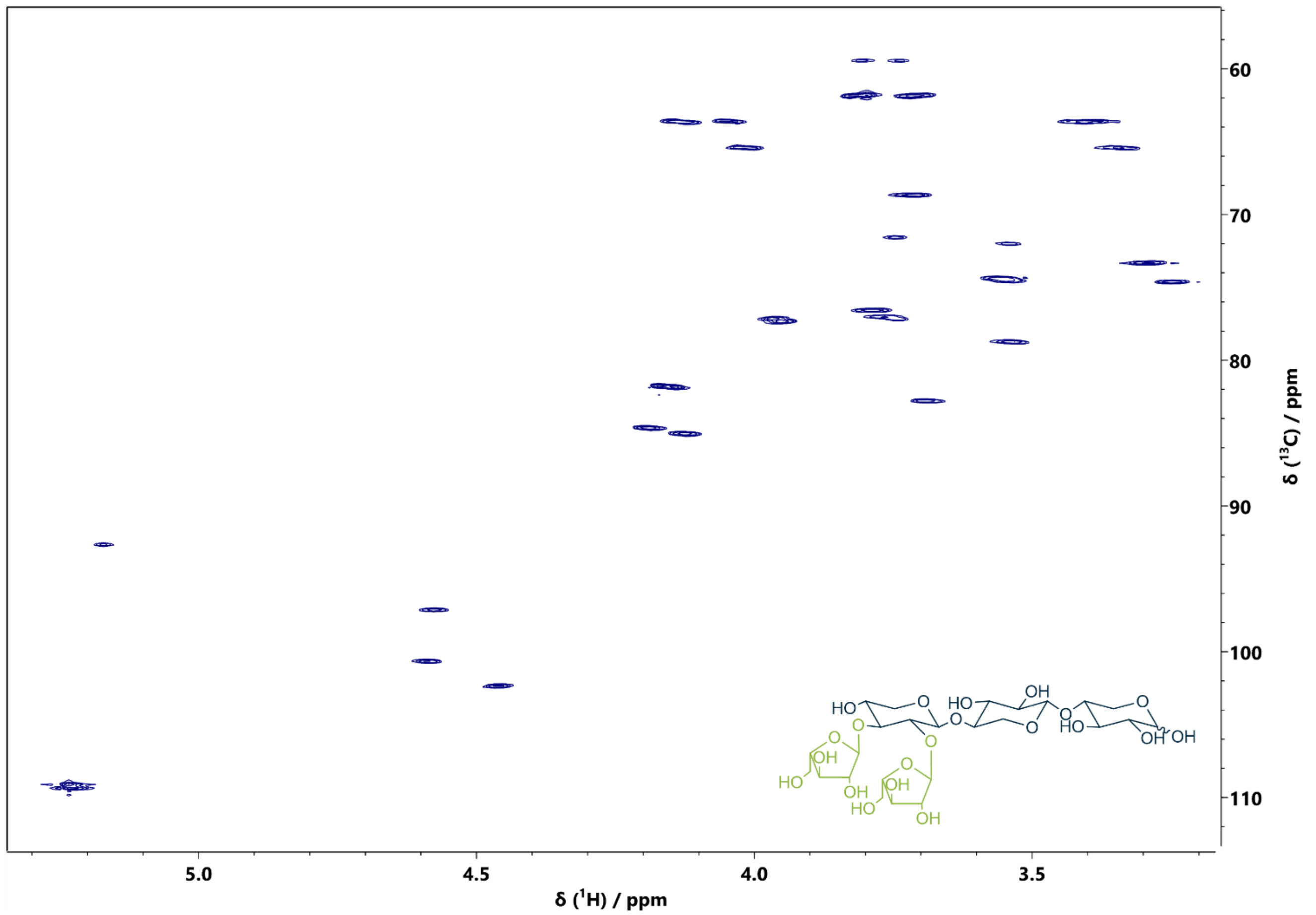

Supplementary Figure 17: HSQC spectrum and chemical structure of the arabinoxylooligosaccharide $A^{2+3}XX$. The acquistion was performed in D_2O using a 500 MHz spectrometer and chemical shifts were referenced against acetone ($\delta_{H/C}$ = 2.22/30.89 ppm). The color scheme of the chemical structure corresponds to **Figure 2**.

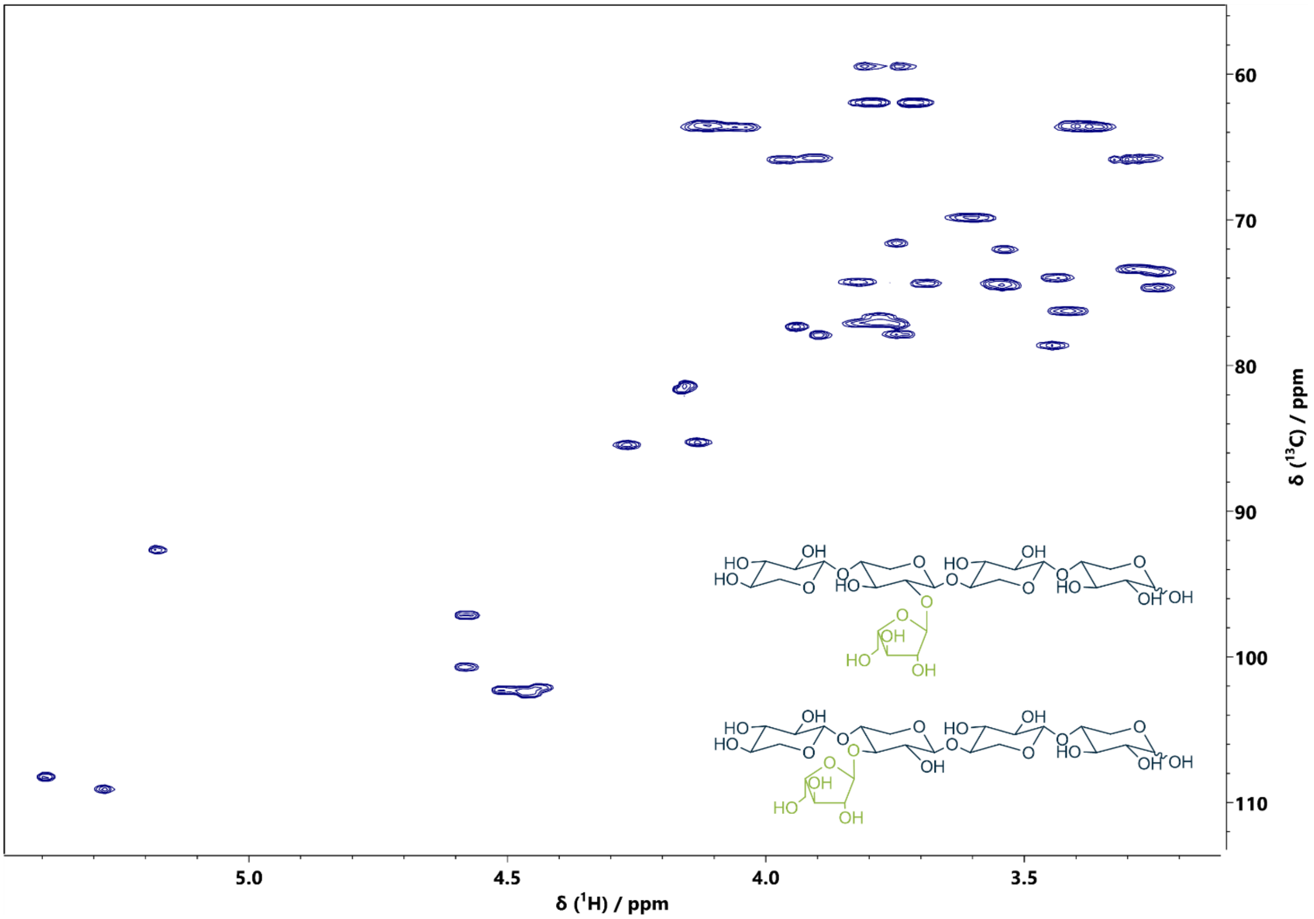

Supplementary Figure 18: HSQC spectrum and chemical structures of the arabinoxylo-oligosaccharides XA^2XX and XA^3XX, which were present in a 47 : 53 mixture. The acquistion was performed in D_2O using a 500 MHz spectrometer and chemical shifts were referenced against acetone ($\delta_{H/C}$ = 2.22/30.89 ppm). The color scheme of the chemical structure corresponds to **Figure 2**.

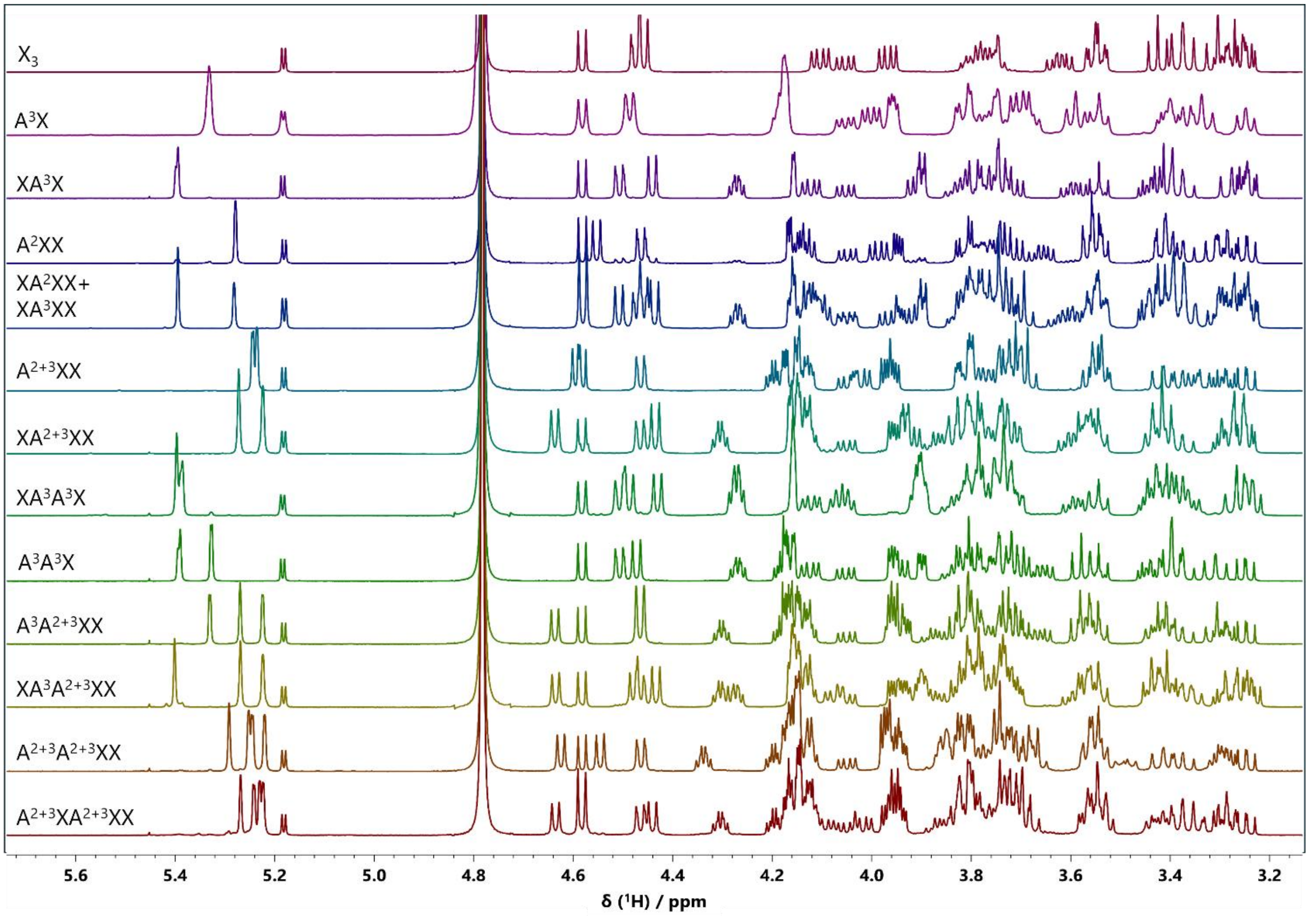

Supplementary Figure 19: Expanded ^{1}H-NMR spectra of selected (isolated) xylo- and arabinoxylo-oligosaccharides partially shown in **Figure 2**. The spectra were recorded in D_2O using a 500 MHz spectrometer and chemical shifts were referenced against acetone (δ_H = 2.22 ppm). The nomenclature of the XOS and AXOS corresponds to the naming system suggested by Faure *et al*., 2009.
